# Active *Cryptococcus neoformans* glucuronoxylomannan production prevents elimination of cryptococcal CNS infection in vivo

**DOI:** 10.1186/s12974-025-03384-9

**Published:** 2025-03-04

**Authors:** Vanessa Enriquez, Melissa E. Munzen, Luz M. Porras, Claudia L. Charles-Niño, Fahong Yu, Karina Alviña, Raddy L. Ramos, Michael R. Dores, Paola Giusti-Rodriguez, Luis R. Martinez

**Affiliations:** 1 Department of Oral Biology, University of Florida College of Dentistry, 1395 Center Drive, DG-48, P.O. Box 100424, Gainesville, FL 32610 USA; 2 Department of Psychiatry, University of Florida College of Medicine, Gainesville, FL USA; 3 Interdisciplinary Center for Biotechnology Research, University of Florida, Gainesville, FL USA; 4 Department of Neuroscience, University of Florida College of Medicine, Gainesville, FL USA; 5 Center for Translational Research in Neurodegenerative Disease, University of Florida, Gainesville, FL USA; 6 McKnight Brain Institute, University of Florida, Gainesville, FL USA; 7 Department of Biomedical Sciences, NYIT College of Osteopathic Medicine, New York Institute of Technology, Old Westbury, NY USA; 8 Department of Biology, Hofstra University, Hempstead, NY USA; 9 Center for Immunology and Transplantation, University of Florida, Gainesville, FL USA; 10 Emerging Pathogens Institute, University of Florida, Gainesville, FL USA

**Keywords:** *C. neoformans*, Cryptococcoma, Meningoencephalitis, Microglia, Phagocytosis

## Abstract

**Background:**

*Cryptococcus neoformans* (*Cn*) causes life-threatening meningoencephalitis in individuals with AIDS. *Cn*’s polysaccharide capsule is mainly composed of glucuronoxylomannan (GXM) and plays a key role in the dysregulation of immunity, resistance to antifungal drugs, and systemic dissemination, including CNS invasion. Although recent studies have begun to elucidate the involvement of microglia in cryptococcosis, our knowledge of these CNS resident phagocytes in the control of cryptococcosis is limited.

**Methods:**

We investigated microglial responses to *Cn* infection and the effect of active capsular production by comparing wild-type H99 and acapsular mutant *cap59* strains using the CX3CR1-EGFP transgenic mouse and a stereotaxic intracerebral infection model.

**Results:**

Microglia had difficulty combating *Cn* H99 infection. Active production and secretion of the capsular material altered the morphology and distribution of microglia around cryptococcomas or fungal brain lesions. It also affected the infiltration of peripheral immune cells to CNS fungal infection. Moreover, RNA sequencing analyses supported the importance of capsule production in immune modulation. Chemotaxis assays demonstrated that active capsular production by *Cn* H99, and especially GXM, impaired microglial motility and fungal phagocytosis.

**Conclusion:**

Our findings suggest that microglia may not be able to control cryptococcal CNS infection and that active capsular production and release may contribute to the progression and persistence of cerebral cryptococcosis.

**Supplementary Information:**

The online version contains supplementary material available at 10.1186/s12974-025-03384-9.

## Introduction

*Cryptococcus neoformans* (*Cn*) is an encapsulated yeast-like fungus that triggers life-threatening meningoencephalitis in both immunocompromised and apparently healthy individuals. Of the approximately 152,000 annual cases of cryptococcal meningoencephalitis (CME) in AIDS victims worldwide, 112,000 resulted in death [1]. *Cn* infects humans when desiccated or poorly encapsulated yeasts/basidiospores are inhaled [2]. Most immunocompetent hosts render *Cn* dormant in their lungs, but immunosuppression can trigger its replication and dissemination via blood or lymph to other organs, especially the brain. During cryptococcosis, fungemia is detected in ∼ 50% of HIV-infected patients [3], and this correlates experimentally with systemic dissemination [4], which is an independent parameter of early mycological failure in humans [3]. *Cn* crosses the blood-brain barrier (BBB) [5] via multiple mechanisms: transcytosis [6], paracellular transit [7], or as “Trojan horse” cargo within host phagocytes [8, 9]. Once inside the brain, *Cn* evades the host immune system and becomes difficult to treat via standard anti-fungal agents [10].

The polysaccharide capsule is a major contributor to *Cn* virulence [11]. The capsule’s principal constituent is glucuronoxylomannan (GXM), which accumulates in the serum and cerebrospinal fluid (CSF; [12, 13]) and specifically enhances *Cn* pathogenesis [14]. Patients suffering cryptococcosis exhibit high GXM levels released around large penetrating vessels in brain tissues studied post-mortem [15]. High GXM levels are associated with many immunosuppressive effects [14], including interference with phagocytosis, antigen presentation, leukocyte migration and proliferation, and specific antibody (Ab) responses. GXM even enhances HIV replication [16]. Moreover, we recently demonstrated that GXM alters endothelial cell tight junction protein expression, weakening the BBB, and promoting *Cn* brain invasion [17].

Microglia are the resident primary immune cells of the CNS associated with *Cn* and its GXM [15] and are potentially critical in CME defense and pathogenesis [18, 19]. Upon exposure to fungal antigens, microglia release cytokines and antimicrobial molecules to recruit CD4^+^ and CD8^+^ T cells, macrophages, and neutrophils that can enter the CNS [20, 21]. Brains of AIDS-associated CME victims exhibit microglia nodules with *Cn*-associated multinucleate giant cells, a histological hallmark of HIV-related encephalitis [22]. Also, microglia localize close to blood vessels during *Cn* BBB transmigration and either engulf or migrate towards the fungi [23]. Despite this, microglia have difficulty in eliminating cryptococci from the CNS independently and do not protect mice with CME [24], possibly due to *Cn*’s ability to form cryptococcomas, a localized brain lesion consisting of a collection of yeast cells entangled in capsular material and characterized by neuronal loss [25]. Cryptococcomas are often surrounded by microglia [26], demonstrating an innate immune response typically observed in infected individuals unable to fight the overwhelming fungal burden. Although microglia are vital in controlling microbial brain tissue colonization, their interactions with *Cn* remain understudied and may be more limited in individuals with defective T cells.

Due to *Cn*’s high associated mortality, treatment challenges, and the high incidence of CME cases, especially for AIDS individuals in sub-Saharan Africa [1], *Cn* was recently placed in the top list of priority fungal pathogens by the World Health Organization. Therefore, comprehensive studies focusing on microglial responses and function against *Cn* are necessary to gain insight on this fungus neurotropism. Here, we used the CX3CR1-Enhanced green fluorescent protein (EGFP) transgenic mouse, which is ideal to study microglia-*Cn* interactions because it drives EGFP expression at the endogenous Cx3cr1 locus, facilitating the visualization of microglia. To assess the importance of active capsular production in cerebral cryptococcosis, we compared microglial and peripheral immune cell responses to wild-type and acapsular mutant *Cn* strains using a recently described mouse model of stereotaxic intracerebral (i.c.) *Cn* infection [27] and microglia-like cells [28]. We also used RNA sequencing (RNA-seq) analysis to identify regulated host genes and biological pathways modulated by the presence or absence of the *Cn* capsule. We demonstrated that *Cn* GXM compromises microglial migration and effector functions, which may facilitate fungal survival and disease progression. Our results may contribute to the development of novel therapeutics and preventive measures for combating and the management of CNS cryptococcosis.

## Results

### High fungal burden in brain tissue of mice infected with encapsulated *Cn* H99 results in death

**Fig. 1:**
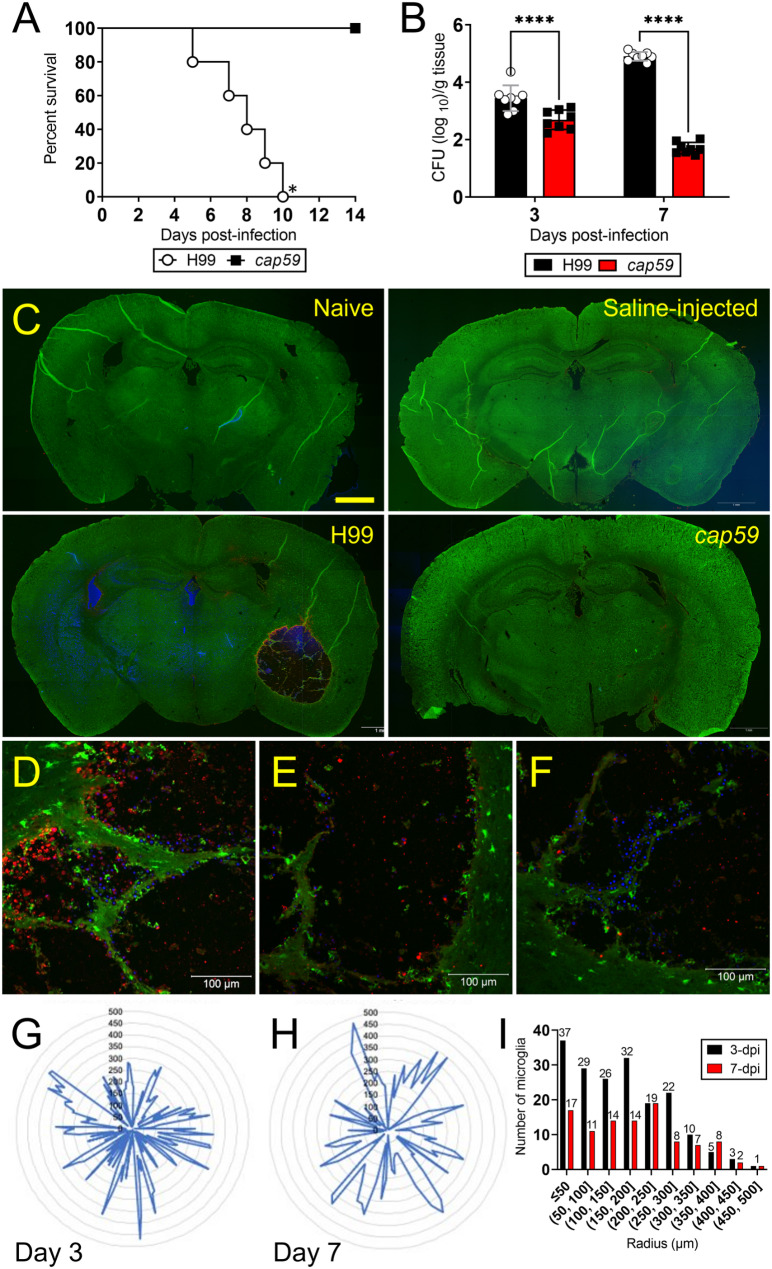
Cryptococcus neoformans (*Cn*) capsular production enhances CX3CR1-enhanced green fluorescent protein (EGFP) mouse mortality by reducing microglial migration to the brain region of infection. (**A**) Survival differences of CX3CR1-EGFP mice (6–8 weeks old) intracerebrally (i.c.) infected with 10^4^ yeast cells of *Cn* strains H99 (wild-type) or *cap59* (acapsular; *n* = 7 per group). Significance (*P* < 0.05) was calculated by log-rank (Mantel-Cox) analysis. Asterisk (*) denotes H99-infected higher mortality compared to *cap59*-infected animals. (**B**) Fungal burden in brains collected from *Cn* H99- or *cap59*-i.c. infected mice with 10^4^ cryptococci (*n* = 8 per group) at 3- and 7-days post-infection (dpi). Bars and error bars denote the mean value and standard deviations (SDs), respectively. Asterisks denote *P*-value significance (****, *P* < 0.0001) calculated using multiple student’s *t*-test analyses. (**C**) Confocal microscopy (CM) of coronal brain tissue sections of naïve, saline-injected, H99-infected, and *cap59*-infected mice (*n* = 4 brains per group) at 3-dpi. EGFP (green) is expressed by CX3CR1-EGFP microglia. Calcofluor white was used to label the cell wall of acapsular or capsular yeast cells (blue). GXM-specific monoclonal antibody 18B7 (mAb 18B7) was used to stain H99 cell capsular- or exo-polysaccharide (red). Scale bar: 1 mm. (**D-F**) High magnification (40X) images show many yeast cells attached to neuronal tissue and considerable capsular accumulation on the edges of the cryptococcoma in the H99-infected mouse. Scale bars: 100 μm. Spatial localization of microglia in relationship to the *Cn* H99-cryptococcoma (**G**) 3- and (**H**) 7-dpi are shown in radar plots. Microglial distances from the cryptococcoma border were measured using NIH ImageJ software and the radius distances plotted. (**I**) The number of microglia per cryptococcoma radii 3- and 7-dpi are shown. This experiment was performed twice, similar results were obtained each time, and all the results combined are presented

We assessed the impact of active *Cn* capsular production on cerebral cryptococcosis by comparing the virulence of wild-type encapsulated H99 and acapsular *cap59* strains. The *cap59* strain is derived from H99, being deficient in GXM export [29]. H99 and *cap59* strains were injected intracerebrally (i.c.) into CX3CR1-EGFP mice (*n* = 7 per group; Fig. 1). Mice infected with H99 cryptococci demonstrated earlier mortality than animals infected with the *cap59* strain (Fig. 1A; [*P* < 0.05; median survival: 8-days post-infection (dpi)]), which survived until the experiment was terminated at 14-dpi. Then, using colony forming units (CFU) determinations, we compared the fungal burden in brains removed from H99- and *cap59*-infected mice (*n* = 8 per group) at 3- and 7-dpi (Fig. 1B). H99-infected brains had significantly higher fungal loads (3-dpi, 3.45 × 10^3^ CFU/g tissue, *P* < 0.0001; 7-dpi, 4.92 × 10^4^ CFU/g tissue, *P* < 0.0001) than those infected with *cap59* (3-dpi, 2.69 × 10^2^ CFU/g tissue; 7-dpi, 1.7 × 10^1^ CFU/g tissue). Similarly, we compared the fungal proliferation of each strain as the infection progressed (3-dpi vs. 7-dpi). Brains infected with H99 and excised at 7-dpi had significantly more CFU numbers than brains removed at 3-dpi (*P* < 0.0001; Fig. 1B). In contrast, *cap59*-infected mice had lower brain fungal burden at 7-dpi than 3-dpi (*P* < 0.0001). These data show that active *Cn* capsule synthesis is essential for fungal virulence and cerebral pathogenesis.

To understand the microglial response during cerebral cryptococcosis that may contribute to the advantage of *Cn* H99 over *cap59* cells to thrive in brain tissue, CX3CR1-EGFP transgenic mice were infected i.c. with 10^4^*Cn* H99 or *cap59* strain cells. Whole-brain coronal tissue sections from naïve (Fig. 1C, upper left), saline injected (Fig. 1C, upper right)- and *cap59* (Fig. 1C, lower right)-infected mice displayed intact architecture and widespread distribution of microglia at 3-dpi. In contrast, tissue sections from H99-infected mice exhibited a large cryptococcoma with a diameter of approximately 1.75 mm (Fig. 1C, lower left). A close inspection of the H99 cryptococcoma evinced considerable tissue damage with activated microglia (Fig. 1D), substantial GXM accumulation at the structural edge [red, GXM-specific monoclonal antibody (mAb) 18B7 (Fig. 1D-E), and considerable number of cryptococci [blue, calcofluor white (CW)] attached to damaged tissue in the innermost areas of the lesion (Fig. 1F). Interestingly, most microglia were localized on the boundaries of the cryptococcoma or on/near damaged tissue instead of inside of the cryptococcoma where most fungi were located. Our findings suggest that *cap59* cells’ deficiency in producing and secreting GXM impairs their ability to form brain cryptocococcoma, while active capsular production by *Cn* H99 facilitates cryptococcoma formation, an environment that is surrounded by vast amounts of GXM and early microglial responses near damaged tissue.

To characterize microglial responses to the cryptococcoma, we measured the distance of individual microglia cell locations from the periphery of the cryptococcoma at 3 (Fig. 1G)- and 7 (Fig. 1H)-dpi. Radar plots were used to trace the distance of a single microglia from the edge of the cryptococcoma (Fig. 1G-H). At 3-dpi, we observed considerable microglial accumulation near the periphery of the cryptococcoma (Fig. 1G), with the majority of the phagocytic cells within less than 200-µm radius from the border (Fig. 1I). At 7-dpi, a sharp reduction in the number of microglia at the border of the cryptococcoma was observed as the cryptococcal infection progressed (Fig. 1H), with most microglia found homogeneously spread within less than 250-µm radius from the edges of the lesion (Fig. 1I). These results demonstrate that microglia recruitment localizes to the cryptococcoma border in early stages of the infection and becomes more diffuse as the disease progresses, potentially making it difficult to eradicate the cryptococcal infection.

### Microglial density and distribution changes during cryptococcal infection

**Fig. 2:**
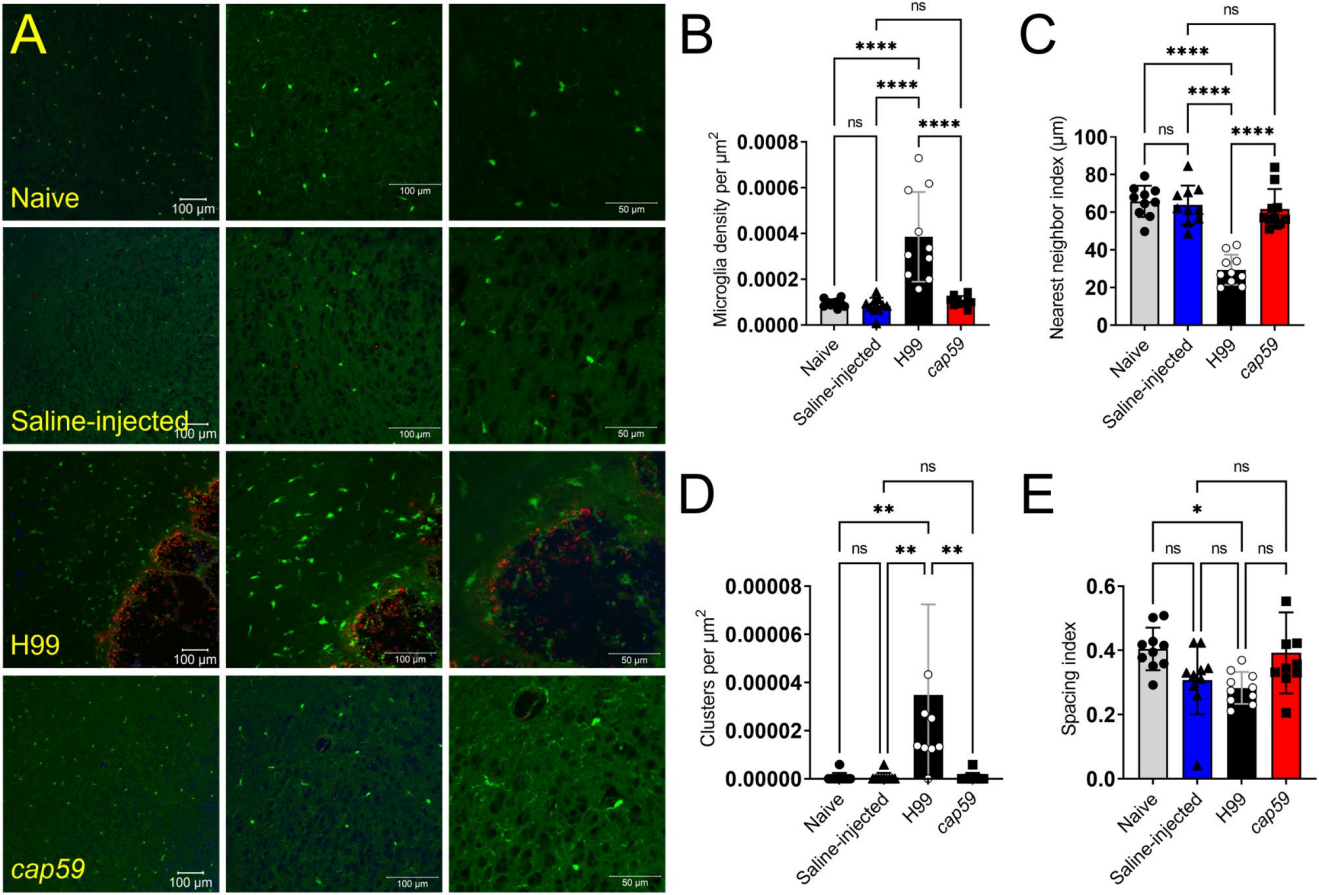
Brains infected with *Cn* strain H99 cells exhibit initial increased activated microglia cell density at 3-dpi. (**A**) Immunofluorescent images of brain tissue sections harvested 3-dpi from naïve, saline-injected, H99-infected, and *cap59*-infected CX3CR1-EGFP mice (*n* = 4 mice per group; 10^4^ fungi). EGFP (green) is expressed by microglia. Calcofluor white was used to label the cell wall of acapsular or capsular yeast cells (blue). GXM-specific mAb 18B7 was used to stain H99 cell capsular- or exo-polysaccharide (red). Left, central, and right panels show 10, 20, and 40X magnification, respectively. Scale bars: 100 μm (10X and 20X) and 50 μm (40X). The 20X images in each group were randomly analyzed (*n* = 10 fields per group) for microglia cell (**B**) density per µm^2^, (**C**) nearest neighbor index (NNI), (**D**) clusters per µm^2^, and (**E**) spacing index (NNI^2^ x microglia cell density) using the ImageJ FIJI software and NNI plugin. For **B-E**, bars and error bars denote mean values and SDs, respectively. Significance (****, *P* < 0.0001; **, *P* < 0.01; *, *P* < 0.05) was calculated by one-way ANOVA and adjusted using Tukey’s *post-hoc* analysis. ns denotes comparisons which are not statistically significant. This experiment was performed twice, similar results were obtained each time, and all the results combined are presented

**Fig. 3:**
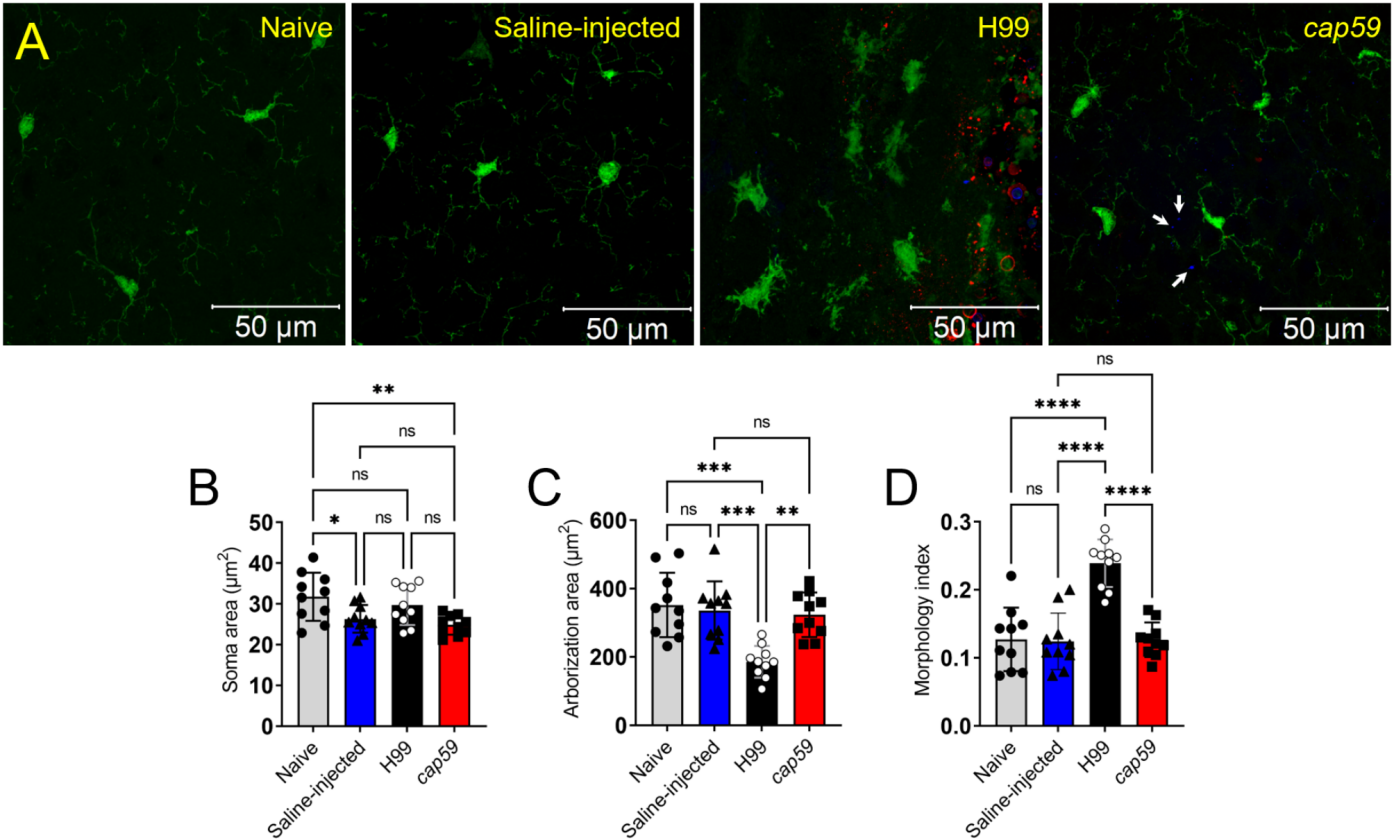
*Cn* H99-infected brains show microglial morphological changes near cryptococcomas. (**A**) CM images of brain tissue sections harvested 3-dpi from naïve, saline-injected, H99-infected, and *cap59*-infected CX3CR1-EGFP mice (*n* = 4 mice per group; 10^4^ fungi). EGFP (green) is expressed by microglia. Calcofluor white was used to label the cell wall of acapsular or capsular yeast cells (blue; white arrows). GXM-specific mAb 18B7 was used to stain H99 cell capsular- or exo-polysaccharide (red). The images were taken at 63X magnification. Scale bars: 50 μm. The images in each group were randomly analyzed (*n* = 10 fields per group) for microglia cell (**B**) soma area, (**C**) arborization area, and (**D**) morphology index (soma area divided by arborization area) using the ImageJ FIJI software. For **B-D**, bars and error bars denote means and SDs, respectively. Significance (****, *P* < 0.0001; ***, *P* < 0.001; **, *P* < 0.01; *, *P* < 0.05) was calculated by one-way ANOVA and adjusted using Tukey’s *post-hoc* analysis. ns denotes comparisons which are not statistically significant. This experiment was performed twice, similar results were obtained each time, and all the results combined are presented

Given the early microglial responses to *Cn* H99 cryptococcomas in the basal ganglia, these lesions were imaged using confocal microscopy (Fig. 2A), and 20X images (middle panels) were analyzed with the ImageJ FIJI software to quantify microglial distribution at 3-dpi (Fig. 2B-E). Similarly, the basal ganglia region of naïve, saline-injected, and *cap59*-infected mice were used as controls. First, the microglial density per micrometer square (µm^2^) was calculated by dividing the number of cells in a random area of tissue near the cryptococcoma or region of injection (Fig. 2B). H99-infected brains evinced significantly higher microglial density than naïve, saline-injected and *cap59*-infected tissue (*P* < 0.0001; Fig. 3B). No differences in microglial density were observed in brain tissues from naïve and saline-injected mice, indicating that tissue damage due to the injection was not a confounding variable affecting microglial distribution. Likewise, differences in microglial density were neither observed between the saline-injected and *cap59*-infected tissue, also suggesting that the acapsular mutant is rapidly cleared during infection.

Then, we determined the nearest neighbor index (NNI) of labeled microglia using the ImageJ FIJI software plugin (Fig. 2C), which is a measurement of the observed mean distance between neighboring cells compared to the expected mean distance in randomly distributed cells. A lower NNI value correlates to cells closer to their neighboring cells. Hence, *Cn* H99-infected brains showed significantly lower NNI than brains removed from all the other groups (*P* < 0.0001). Naïve, saline-injected, and *cap59*-infected tissues showed comparable NNI values. These findings indicate that encapsulated *Cn* attracts a higher density of microglial cells to the site of infection.

Next, we determined and compared the number of microglial clusters in naïve, saline-injected, H99-infected, and *cap59*-infected tissue (Fig. 2D). We arbitrarily standardized our analysis and established that any NNI value less than 12-µm was considered a single cluster. The number of clusters was divided by the area of the region of interest (ROI) to calculate the number of clusters per µm^2^. The density of microglial clusters in H99-infected brains was significantly higher than those observed in naïve, saline-injected, and *cap59*-infected brains (*P* < 0.01). There were no differences in the number of microglial clusters per µm^2^ between the uninfected and the acapsular mutant-infected mice.

Finally, we measured the spacing index among microglia from brains excised from mice in each group (Fig. 2E). This parameter measures the distribution of microglia in an area of tissue [30] and was calculated by taking the square of the average NNI divided by the microglial density. The spacing index of microglia in tissue from *Cn* H99-infected brains was only significantly lower than in tissue from naïve brains (*P* < 0.05), although no differences were observed when compared to saline-injected and *cap59*-infected mice. Similarly, there were no statistical differences between the spacing indices of naïve, saline-injected, and *cap59*-infected brains.

These results indicate *Cn* H99 infection alters the density and distribution of microglia near the cryptococcoma, highlighting the importance of active capsular polysaccharide production in microglial responses to the fungus.

### *Cn* H99 causes microglial morphological changes

To understand the association between microglial function/responses and morphological changes to cryptococcal infection, especially in presence and absence of the capsule, we imaged and analyzed the morphology of microglia upon infection with *Cn* H99 or *cap59* in brain tissue cross-sections at 3-dpi (Fig. 3). Confocal microscopy images of microglia displayed a ramified morphology in naïve, saline-injected, and *cap59*-infected brains (Fig. 3A). In contrast, microglia in H99-infected brains exhibited amoeboid shape around the cryptococcoma. To quantitatively evaluate the morphology of microglia near the striatal injection/infection region (Fig. 3B), we measured the cell soma and arborization area and calculated the morphology index [30]. The soma area was determined by delineating the periphery of each cell soma. There were no differences in the soma area between H99-infected mice and the other groups. However, saline-injected (*P* < 0.05) and *cap59*-infected (*P* < 0.01) microglia showed significantly less soma area than cells in naïve brains. To measure the arborization area (Fig. 3C), a polygon outline was created around individual cells by designating the tip of each branch as a vertex to represent the extent of the branching area covered. Compared to the naive, saline-injected, and *cap-59*-infected brains, the microglia in H99-infected tissues demonstrated a significant reduction in arborization area. Furthermore, the morphology index was calculated by dividing the soma area by the arborization area, a ratio used to inform overall morphological changes (Fig. 3D). Due to the reduced arborization area, H99-infected brains had a significantly higher microglial morphological index compared to the naive, saline-injected, and *cap59*-infected brains (*P* < 0.0001). No differences in the microglial morphological index were observed when naïve, saline-injected, and *cap59*-infected brains were compared. These findings revealed that encapsulated *Cn* H99 cells stimulate microglial morphological alterations, which may be critical for infection progression and disease control.

### Active capsular production by *Cn* inhibits the migration of microglia

**Fig. 4:**
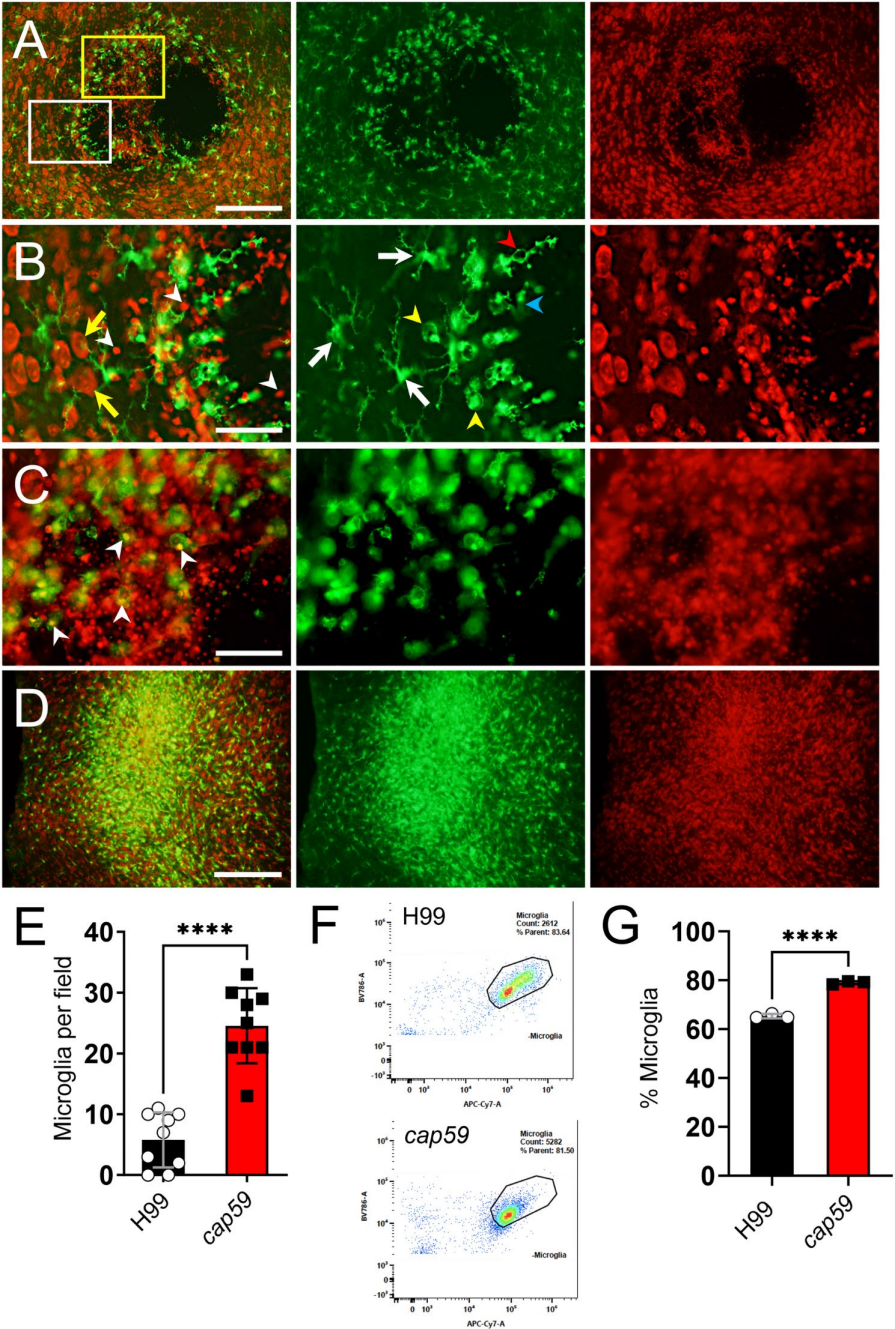
Active capsular production by *Cn* reduces microglial cell migration. (**A**) Immunofluorescent images of basal ganglia tissue sections harvested at 7-dpi from a CX3CR1-EGFP mouse infected i.c. with *Cn* H99 (*n* = 4 brains per group; 10^4^ fungi). EGFP (green) is expressed by microglia. Propidium iodide was used to label fungi and neurons. (**B**) High magnification image (63X; white rectangle area in **A**) of the cryptococcoma border shows yeasts (white arrowheads) interacting with microglia and neuronal tissue (yellow arrows). Center panels show ramified (white arrows), phagocytic or amoeboid (yellow arrowhead), dystrophic (light blue arrowhead), and rod-shaped (red arrowhead) microglia. (**C**) High magnification image (63X; yellow rectangle area in **A**) of the central region of the cryptococcoma shows abundant cryptococci surrounded by mainly phagocytic microglia. White arrowheads denote phagocytosed cryptococci. Scale bar: 200 μm. (**D**) Massive migration of microglia to the area of *cap59* infection at 7-dpi. (**E**) Quantification of microglia per field (*n* = 9 fields per group) in H99 or *cap59*-infected tissue at 7-dpi. These experiments were performed twice, similar results were obtained each time, and all the results combined are presented. (**F**) Representative flow cytometry dot plots for microglia in basal ganglia infected with H99 (upper panel)- or *cap59* (lower panel) cryptococci at 7-dpi are shown. (**G**) Percentage (%) of microglia in brain tissue infected with H99 or *cap59* cells at 7-dpi. Each symbol represents an independent replicate (*n* = 3) where ≥ 10,000 events per group were measured. For **E** and **G**, bars and error bars denote mean values and SDs, respectively. Significance (****, *P* < 0.0001) was calculated by student’s *t*-test analysis

Since microglia disperse away from *Cn* H99 cryptococcomas as the infection progresses, CX3CR1-EGFP mice were also i.c.-infected with H99 or *cap59* cryptococci and their brain tissues were imaged with confocal microscopy at 7-dpi. EGFP-expressing microglia and propidium iodide-stained *Cn* and neurons are shown in green and red, respectively (Fig. 4A-D). Brains infected with H99 cells evinced cryptococcoma formation (Fig. 4A-C) and reduced microglia per field (*n* = 9 per group; mean: 5.8 cells per field; Fig. 4E). A closer view (white rectangle) at the periphery of the H99 cryptococcoma demonstrated abundant ramified microglia (white arrows) closer to neurons (yellow arrows) whereas different morphological states were observed in microglia closely interacting with cryptococci (white arrowheads) including phagocytic or amoeboid (yellow arrowheads), rod-shaped (red arrowhead), and dystrophic (blue arrowheads) cells (Fig. 4B). Another region of the cryptococcoma (yellow rectangle) shows considerable accumulation of cryptococci surrounded by high density of phagocytic microglia (Fig. 4C). White arrowheads denote microglia with engulfed cryptococci. In contrast, brain tissue infected with *cap59* cells revealed a drastic increase in phagocytic/amoeboid microglial infiltration per field (mean: 24.6 cells per field; *P* < 0.0001; Fig. 4D-E). Additionally, we performed flow cytometry in brain tissue to quantify the percentage (%) of microglial infiltration in infected tissue with *Cn* H99 or *cap59* cells and confirmed the results obtained by confocal microscopy (Fig. 4F-G). The flow cytometry gating strategy utilized is shown in SFig. 1. Representative dot plots (Fig. 4F) showed that H99-infected brains (66.4%) had lower microglial infiltration on the site of infection than *cap59*-infected brains (79.6%; *P* < 0.0001; Fig. 4G). We further determined the involvement of other immune cells in the microglial response to *Cn* H99 or *cap59* infection (SFig. 2). Neutrophils (8.06% vs. 1.13%; *P* < 0.0001; SFig. 2 A), monocytes (1.09% vs. 0.57%; *P* < 0.01; SFig. 2 C), dendritic cells (2.57% vs. 0.82%; *P* < 0.01; SFig. 2D), and CD4^+^ T cells (1.26% vs. 0.36%; *P* < 0.001; SFig. 2E) were significantly increased in brains infected with *Cn* H99 compared to *cap59* cells. In contrast, macrophages (5.11% vs. 8.47%; *P* < 0.001; SFig. 2B) and B or B220^+^ cells (3.31% vs. 33.44%; *P* < 0.0001; SFig. 2E) showed less infiltration in brains infected with H99 cells than in those infected with *cap59* cells. Of note, there may be a subpopulation of B220-APC-A^+^/CD11b-APC-Cy7^+^ or F4/80-BV711^+^ present in the *cap59-*infected mice brains that result in potentially inflated cell frequency percentages. To explain this occurrence with the flow cytometry gating strategy outlined previously [31], it is possible that a peripheral B220-APC-A^+^ progenitor cell population distinct from B cells may be recruited to the CNS and differentiate into phagocytic cells such as macrophages or microglia during *cap59* infection [32], resulting in enhanced clearance of *cap59* from the brain. While we did not stain CD34 to label progenitor cells in this study, it will be a necessary parameter to account for in future studies to determine the exact nature of this finding. There were no differences in the brain tissue recruitment of CD8^+^ T cells in the groups compared (SFig. 2E). These results demonstrate the inability of the acapsular mutant cells to form cryptococcoma possibly due to a massive microglial response and infiltration as well as considerable support of peripheral macrophages and B cells, suggesting that active capsular production is required for brain tissue colonization.

### Overall transcriptomic profiles vary during *Cn* infection

**Fig. 5:**
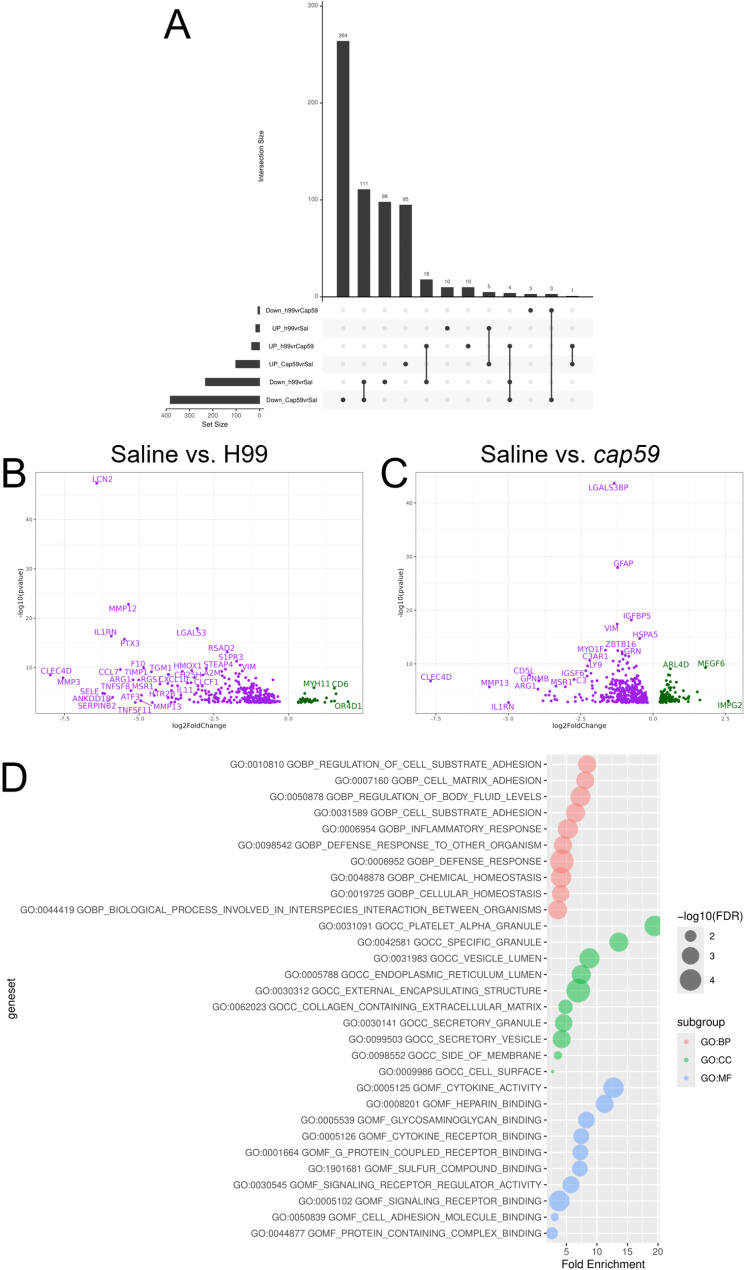
Active *Cn* capsular production induces extensive transcriptomic and biological changes in the mouse brain. C57BL/6 mice (6–8 weeks old; *n* = 3 animals per group) were i.c. infected with 10^4^*Cn* strains H99 or *cap59* cells and euthanized at 7-dpi. Naïve and saline-injected mice were used as controls. RNA extraction, library preparation, and sequencing using the Illumina NovaSeq 6000 platform were performed from brain tissue samples. (**A**) Upset plot of DEGs up- or down-regulated across conditions. Set size = total number of DEGs per condition. Interaction size indicates the number of DEGs that are unique (dots) or that overlap across conditions (dots linked by line). Volcano plots displaying differentially expressed genes (DEGs; false discovery rate or q value < 0.05) for (**B**) H99-infected vs. saline-injected animals and (**C**) *cap59*-infected vs. saline-injected animals. Downregulated genes appear in purple, while upregulated genes appear in green. (**D**) Bubble plots of gene ontology (GO) analysis using hypergeometric tests (Bonferroni-corrected hypergeometric *P* < 0.05) of DEGs (FDR < 0.10) comparing *cap59* and H99 at 7-dpi. The background gene set was all protein coding genes detected in the experiment. Any gene set with less than 5 genes, after filtering out genes not in the background, was excluded

Given the observed structural and morphological microglial changes, we sought to determine how infection with the capsular *Cn* H99 strain impacted gene expression in brain tissue at 7-dpi using RNA-seq. Naïve and saline-injected brain tissues were also included to control for tissue damage due to the injection. Following quality control, principal component analysis demonstrated minimal variance between naïve- and saline-injected samples, clear separation between naïve- and saline-injected and *cap59*-infected and *Cn* H99-infected samples, and 9% variance in the expression profiles of *cap59*-infected and *Cn* H99-infected samples (SFig. 3 A). Hierarchical clustering of RNA seq data showed that naïve and saline-injected samples clustered closely together, while *Cn* H99- and *cap59*-infected samples formed a separate cluster, with *cap59*-infected samples falling closer to saline-injected samples (SFig. 3B). To compare the expression profiles between the four experimental conditions, we performed differential gene expression using DESeq2 [33] using a false discovery rate of q < 0.05 (Fig. 5). Down- and up-regulated genes were examined separately to facilitate interpretation of findings. Intersections between differentially expressed genes (DEGs) across conditions were analyzed using UpSet analysis [34] (Fig. 5A). *Cn* H99-infected brains displayed 15 up- and 231 down-regulated DEGs compared to saline-injected animals (Fig. 5B; STable 1); 5 of the up-regulated and 111 of the down-regulated DEGs overlapped across the two treatment groups (STable 2). There were 382 down- and 102 up-regulated DEGs in *cap59*-infected compared to saline-injected animals (Fig. 5C; STable 3).

Hypergeometric tests of DEGs (FDR < 0.1) identified enriched Gene Ontology terms (GO; *P* < 0.05; [35, 36]) for each comparison group. *Cn* H99 downregulated DEGs were enriched for terms related to infection and immune response as reflected in the top categories within the biological process (GO: BP; response to infection, regulation of inflammatory response, and positive regulation of cytokine production), cellular compartment (GO: CC; platelet al.pha granule, complex of collagen trimers, and protein complex involved in cell adhesion), and molecular function (GO: MF; pathogen associated molecular pattern receptor activity, NAD+-protein ADP-ribosyltransferase activity, and proteoglycan binding) terms (SFig. 4). Downregulated DEGs after *cap59* infection were enriched for terms related to immune cells and response across the biological process (GO: BP; leukocyte activation, adhesion, and migration, innate immune response, and cytokine production), cellular compartment (GO: CC; lysosomal lumen, phagocytic vesicle, and vacuolar lumen), and molecular function (GO: MF; Toll like receptor activity, immune receptor activity, and cytokine binding and receptor activity) (SFig. 5). On the other hand, upregulated DEGs after *cap59* infection were enriched for terms related to neuronal parts and function as evidenced in the outputs of the biological process (GO: BP; cholinergic synaptic transmission, regulation of behavior, negative regulation of ion transporter activity, and response to dopamine), cellular compartment (GO: CC; calcium channel complex, synaptic vesicle membrane, and cation channel complex), and molecular function (GO: MF; calmodulin binding, neurotransmitter activity, and calcium ion transmembrane transporter activity) ontologies (SFig. 6).

Shared ontologies for downregulated genes were extensive (SFig. 7 A) and included terms related to inflammatory response, positive regulation of cytokine production, neutrophil degranulation, and leukocyte activation, while upregulated genes displayed shared ontologies for cAMP signaling pathway and vascular muscle contraction (SFig. 7B). PPI network analysis of downregulated genes included processes related to positive regulation of response to external stimuli, microglia pathogen phagocytosis, and extracellular matrix organization (SFig. 8). Finally, tissue/cell-type-specific analysis of downregulated DEGs using the PaGenBase database (Metascape) evidenced an enrichment for unstimulated macrophages, microglia, and osteoclasts (SFig. 9).

When comparing the two infected groups against each other, there were 33 up- and 6 down-regulated DEGs in *cap59*-infected compared to H99-infected animals (STable 2). Thus, enrichment analysis suggests that H99 infection downregulates biological processes related to positive regulation of vasculature development, leukocyte chemotaxis, and regulation of cell substrate adhesion; cellular compartment terms related to platelet alpha granule and vesicle lumen; and molecular function terms related to cytokine activity, heparin binding, and glycosaminoglycan binding compared to *cap59*-infected animals (Fig. 5D). These findings demonstrated (and validated previous literature) that *Cn* H99 capsular polysaccharide strongly modulates CNS immunity, worsening disease outcomes.

### Active *Cn* H99 capsular production reduces NR-9460 microglial cell responses

**Fig. 6:**
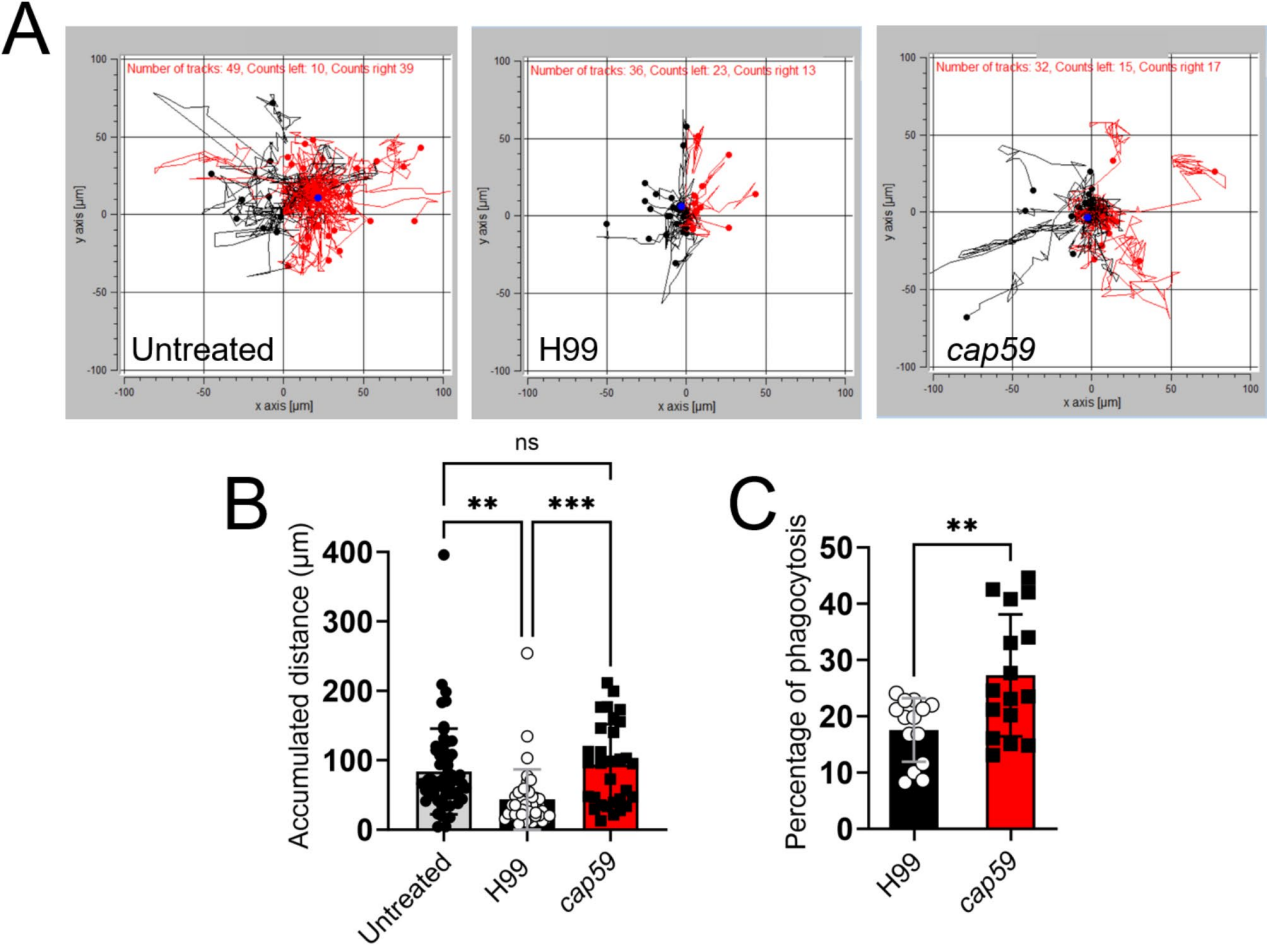
Active production of capsular polysaccharide by *Cn* H99 reduces microglia-like cell migration and cryptococcal phagocytosis. (**A**) Representative individual NR-9460 microglia-like cell migration tracks recorded after incubation in absence (untreated) or with *Cn* H99 or *cap59* cells for 16 h. Microglia were seeded at a density of 3 × 10^6^ cells/mL and *Cn* was added at a 10 (fungi):1 (microglia) ratio. The black and red tracks represent net leftward and rightward movements, respectively. (**B**) The accumulated distance of NR-9460 cells incubated without (untreated; *n* = 108 cell tracks) or with H99 (*n* = 78 cell tracks) or *cap59* (*n* = 60 cell tracks) cryptococci was calculated using the ImageJ FIJI Manual Tracking plugin and Chemotaxis and Migration tool software. Bars and error bars denote mean values and SDs of individual microglia cell tracks, respectively. Significance (***, *P* < 0.001; **, *P* < 0.01) was calculated by one-way ANOVA and adjusted using Tukey’s *post-hoc* analysis. ns denotes comparisons which are not statistically significant. (**C**) The number of phagocytosed H99 or *cap59* cryptococci per microglia-like cell (*n* = 14 fields per group; ≥ 100 microglia per field) after incubation for 4 h was determined. Bars and error bars denote mean values and SDs, respectively. Significance (**, *P* < 0.01) was calculated by student’s *t*-test analysis. These experiments were performed twice, similar results were obtained each time, and all the results combined are presented

To investigate the effect of *Cn* active capsular production on the functional activity of microglia and given the inhibition of microglial responses against *Cn* H99 cells in vivo, we compared the ability of murine NR-9460 microglia-like cells to migrate and phagocytose the H99 and *cap59* strains (Fig. 6). For the migration assays, NR-9460 cells were incubated alone or with cells of either cryptococcal strain for 16 h and their movement were documented using live-microscopy. Each cell movement was tracked to generate migration paths that were transformed into migration distances (Fig. 6A). The untreated control and *cap59*-incubated microglia exhibited random and substantially dispersed migration on the grid relative to H99-incubated microglia, which show localized and restricted movements to the area of initial inoculation (Fig. 6A). In this regard, we measured the accumulated distance migrated by cells in each condition (Fig. 6B). *Cn* H99-incubated microglia (*n* = 78 cells, mean: 43.4 μm) evinced significantly lesser accumulated movement over time compared to untreated (*n* = 108 cells, mean: 84 μm; *P* < 0.01) and *cap59*-incubated (*n* = 60 cells, mean: 93.9 μm; *P* < 0.001) cells. For the phagocytosis assay, *cap59* (*n* = 16 replicates per group; mean: 27.3%) cells were significantly more engulfed by microglia than H99 (mean: 17.6%, *P* < 0.01) cells (Fig. 6C). These observations demonstrate that active *Cn* capsular production compromises microglial responses and effector functions, which may facilitate CNS infection and fungal persistence.

### *Cn* GXM impairs microglia-like cell migration and fungal phagocytosis

**Fig. 7:**
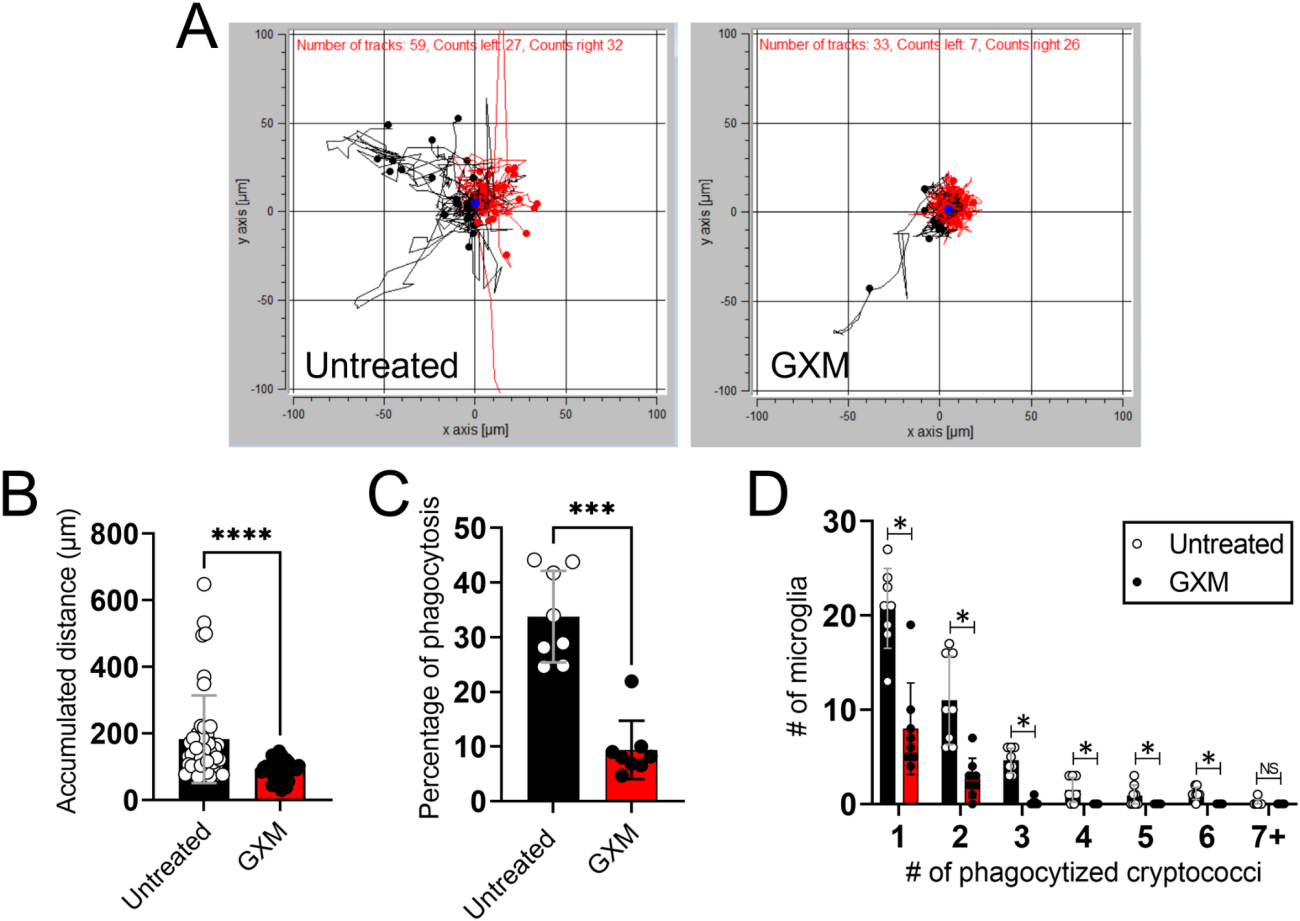
*Cn* glucuronoxylomannan (GXM) reduces microglia-like cell migration and cryptococcal phagocytosis. (**A**) Representative individual microglia-like cell migration tracks recorded after incubation in absence (untreated) or with 10 µg/mL GXM purified from *Cn* H99 for 16 h. Microglia were seeded at a density of 3 × 10^6^ cells/mL. The black and red tracks represent net leftward and rightward movements, respectively. (**B**) The accumulated distance of NR-9460 cells incubated without (untreated; *n* = 59 cell tracks) or with GXM (*n* = 33 cell tracks) was calculated using the ImageJ FIJI Manual Tracking plugin and Chemotaxis and Migration tool software. (**C**) The percentage of cryptococcal phagocytosis by NR-9460 cells incubated with or without GXM was determined (*n* = 8 fields per group; ≥ 100 microglia per field) using the Giemsa staining. (**D**) The number of phagocytosed cryptococci per microglia-like cell (*n* = 8 fields per group; ≥ 100 microglia per field) after exposure to 10 µg/mL GXM for 2 h was assessed. For **B-D**, bars and error bars denote mean values and SDs, respectively. Significance (****, *P* < 0.0001; ***, *P* < 0.001; *, *P* < 0.05) was calculated by individual (**B-C**) and multiple (**D**) student’s *t*-test analyses. ns denotes comparisons which are not statistically significant. These experiments were performed twice, similar results were obtained each time, and all the results combined are presented

GXM is the main constituent of *Cn*’s capsule, contributing extensively to *Cn* pathogenesis because this polysaccharide accumulates in serum, CSF, and tissues while causing severe immunity defects. Since active capsular production by *Cn* dysregulates microglial responses, we further investigated the specific impact of a physiological GXM (10 µg/mL) concentration on microglial migration and fungal phagocytosis (Fig. 7). GXM-treated NR-9460 microglia-like cells evinced reduced motility compared to untreated cells (Fig. 7A). In fact, GXM-treated microglia (*n* = 33 cells, mean: 93.2 μm; *P* < 0.0001) displayed significantly smaller accumulated distance than untreated cells (*n* = 59, mean: 183.4 μm; Fig. 7B). Additionally, GXM exposure significantly reduced cryptococcal phagocytosis by microglia relative to untreated cells (*n* = 8 per group; *P* < 0.001; Fig. 7C). GXM-treated microglia took up less cryptococci per cell compared to untreated cells (*P* < 0.05; Fig. 7D). These data validate that GXM dysregulates microglial motility and cryptococcal engulfment suggesting that the secretion and deposition of this polysaccharide may challenge host responses to infection.

## Discussion

We demonstrated that active production of *Cn*’s capsular polysaccharide is necessary for fungal survival in the CNS and the progression of cerebral cryptococcosis. Mice infected with capsular H99 cryptococci in the brain evinced cryptococcoma formation, higher fungal load, and a 100% mortality by 10-dpi. Interestingly, H99-cryptococcomas exhibited cryptococci with accumulation of GXM in the periphery. Microglia were observed near these brain lesions at 3-dpi, although these CNS resident phagocytes dispersed away from the cryptococcoma as the infection progressed (7-dpi). We previously established that microglia within the cryptococcoma environment become activated, however in regions of damage surrounding the cryptococcoma these phagocytes become dystrophic and necrotic [27]. Here, the microglial diffusion away from the cryptococcoma as the H99-infection progressed raises the possibility that microglial infiltration may become more localized in this later stage of infection to avoid regions of tissue necrosis. It is also possible that *Cn* infection and capsular polysaccharide secretion induce microglial cell death. Chiapello and colleagues demonstrated that *Cn* infection induces apoptosis in lungs and spleen of rats and GXM causes lymphocyte apoptosis in vitro [37]. In addition, the *Cn* capsular polysaccharide component galactoxylomannan or GalXM triggers apoptosis of human T cells via caspase-8 activation [38]. Alternatively, the reduced infiltration observed in the later stage of infection may be explained by the altered gene expression profile in *Cn* H99*-*infected mouse brains. Our transcriptomics data revealed downregulated genes associated with leukocyte activation and migration, innate immune responses, and cytokine production at 7-dpi, suggesting that accumulation of H99 in a cryptococcoma dysregulates the immune response and limits signaling for microglial activation and migration towards the lesion.

As expected, acapsular mutant *cap59* cells were avirulent in brain-infected mice due to the absence of the capsule and their inability to proliferate and form cryptococcomas. It has been established that microglia produce chemokines including CCL3/macrophage inflammatory protein-1α, CCL2/monocyte chemoattractant protein-1, and IL-8, and this production is reduced when stimulated with capsular polysaccharide [21]. Since microglia are a major source of pro-inflammatory and chemotactic cytokines [39], the presence of the acapsular fungus and lack of GXM explains the activation and massive migration of these CNS resident phagocytes during infection and their potential role in facilitating the control and rapid elimination of *cap59* cells. As previously shown in vitro, *cap67* yeasts [40], a mutant with defective capsule production derived from *C. deneoformans* B3501 (formerly *C. neoformans* var. *neoformans* or serotype D) strain, were more susceptible to phagocytosis and killing by BV-2 microglia than the parental encapsulated cells. However, *C. deneoformans* invades the brain in much lower rates than *C. neoformans* (formerly *neoformans* var. *grubii* or serotype A), suggesting that our studies not only complement these published results but also were performed in a cryptococcal species that it is most likely to invade the brain. Microglial contribution to the eradication of *cap59*, though not directly observed in vivo in this study, may be enhanced by the inability of this mutant strain to modulate the pH of the phagosome and avoid killing [41], which otherwise can be altered by the cryptococcal capsule to enable fungal survival [24]. Notably, *cap59* cells, although reduced in infected brains at 7-dpi, were not completely cleared from the brain. It is conceivable that complete clearance of the acapsular cells from brain tissue occurs further post-infection or that those cells not eliminated by microglia or peripheral cells moved to the subarachnoid space and ventricular system and are floating in the CSF, avoiding detection by our CFU determination or microscopic assays.

Saline-injected and naïve brains showed similar microglial and tissue inflammatory responses, which are consistent with inflammation and tissue healing observed in murine models of spinal cord [42] and focal traumatic brain injury [43]. Although not a natural *Cn* infection route, these results indicate that our i.c. infection model [27] allows us to directly study CME progression with minimal immune response alterations associated with the injection. Further, stereotaxic injections are extensively used in neuroscience research and are the safest and best suited method to study infectious disease processes in the CNS including cryptococcosis [27, 44]. We also used the i.c. infection model over intranasal or intratracheal (pulmonary) and intravenous (systemic) models due to the resulting focal localization of cryptococcomas in the mouse brain that resemble human lesions. In fact, i.c. H99-infected mice show similar CME manifestations as described in human patients [15, 18]. Moreover, these infected mice develop distinctive cerebral cryptococcosis stages including edema, intracranial pressure, and hydrocephalus [27], making it an excellent model to dissect the direct neurotoxic impact of *Cn* infection, GXM release, and disease progression. In addition, while both pulmonary and systemic infections also show comparable fungal brain pathology, major differences include the high number of cryptococcomas and their wide brain dissemination. These observations severely compromise our ability to study the impact of *Cn* infection or virulence factors on specific brain regions and their specifically related cognitive and motor functions.

We recently demonstrated that cryptococcal brain infection changes microglia morphology and described different phenotypes including activated or hypertrophic, dystrophic, phagocytic or amoeboid, rod-shaped, and ramified or homeostatic [28]. Our confocal images of the interactions of *Cn* and microglia in the cryptococcoma show the presence of microglia in all these phenotypes and provide evidence for future single cell analysis to determine the function of each of these morphologies in the setting of neurocryptococcosis. Given their remarkable phenotypic plasticity and the limited understanding on microglial interactions with *Cn*, we further combined microscopy, image analysis, and spatial statistical techniques to characterize microglia morphological changes and distribution around the cryptococcoma [45]. Our data show that at 3-dpi, microglia responses to local cryptococcal basal ganglia infection resulted in moderate microglia recruitment to the site of infection as demonstrated by the increased density and clustering of EGFP^+^ cells and a more closely packed yet regularly spaced organization around the brain lesion, as evidenced by the measures of NNI and spacing index (especially vs. naïve). In addition, microglial activation near H99-formed cryptococcomas was associated with a decrease in arborization and an increase in morphology index, reflecting the morphological transition from a ramified or homeostatic to an activated amoeboid-like phenotype. Notably, no major changes in microglial morphology and distribution were observed in naïve, saline-injected, and *cap59*-infected brain tissue. These results suggest that the injection involving the i.c. mouse model does not alter microglial responses considerably, therefore validating this model for future studies of these CNS resident phagocytes in the setting of cryptococcal infection. Additionally, this data corroborates that capsular production is required for cryptococcal survival and persistence in the brain.

Because we observed distinct microglial responses to *Cn* H99 and *cap59* cells at 7-dpi, we performed brain tissue immunoprofiling using flow cytometry. Our findings validated the confocal microscopy results and demonstrated that brains infected with *cap59* cells had higher microglial number than *Cn* H99-infected brains, suggesting that the absence of the capsule makes acapsular cells more susceptible to elimination by CNS-resident or infiltrating phagocytes. Interestingly, brains infected with the acapsular mutant also showed high infiltration of B cells and peripheral macrophages. Considering that adaptive immunity against *Cn* in mice occurs between 4 and 7-dpi, it is possible that *cap59* cells activate B cells [46], which become plasma cells, and secrete fungal-specific Abs that promote extensive acapsular cell phagocytosis by microglia and macrophages. This hypothesis is supported by our in vitro results demonstrating that *cap59* cells are more phagocytosed in presence of mAb18B7 by microglia-like cells than *Cn* H99 cells. B cell responses and Ab production deficiency have shown to increase a risk for developing cryptococcal infections in both immuno-competent [47] and -compromised [48] patients. Brain-infected with *Cn* H99 showed a 10-fold reduction in B cell infiltration, thus indicating the importance of these lymphocytes and their immunoglobulins even in an immunocompetent host considering that CD4^+^ T cell responses were stronger in these animals. In this regard, CME has been associated with lower IgG production in non-HIV individuals with normal T cell counts [47] and with X-linked hyper IgM syndrome [49], which is characterized by lower IgG, IgA and IgE. Similarly, the reduction in migration of peripheral macrophages to the CNS during *Cn* H99 infection is potentially linked to the impaired B cell responses, although further studies to confirm this association are needed. Neutrophils, monocytes, and dendritic cells infiltrated significantly higher in *Cn* H99- than *cap59*-infected brains. Neutrophils are actively recruited to the CNS during viral-induced encephalitis [50, 51]. In mice with cerebral cryptococcosis, depletion of neutrophils augments brain fungal burden since these granulocytes recognize *Cn* via complement C3 and aid in cryptococcal CNS clearance [52]. Likewise, *Cn* brain infection can cause increased monocyte [53] and dendritic cell [54] recruitment. However, even though the *Cn* H99-infected brain doubled the percentage of monocytes and dendritic cells compared to those infected with *cap59*, the number of monocytes and dendritic cells in brains from mice infected with H99 was relatively low or approximately 1–2% of all the immune cells studied, which potentiates an active role of microglia in combating the infection. Furthermore, our immunoprofiling data from brains infected with either *Cn* H99 or *cap59* strains also suggests that the observed increase in the number of microglia is due to proliferation and not to monocyte differentiation.

Our transcriptomic analysis indicates that the chemokine genes *Ccl2*, *Ccl7*, and *Ccl12* are downregulated in mice infected with H99. CCR2 is activated by CCL2, CCL7, CCL8 and CCL12 (in mice)/CCL13 (in humans) [55]. Our findings support previous evidence indicating that CCR2 signaling stimulates inflammatory monocyte infiltration into the CNS and contributes to the progression of CME in mice [56]. Matrix metalloproteinase-12 (MMP-12), which is also downregulated in H99-infected brains, modulates CCL2 production in pulmonary inflammation against *Cn* [57], indicating its important role in the immune response to combat the fungus. Lipocalin 2 (*Lcn2*) is a siderophore-binding protein involved in cellular iron transport and neuroinflammation. However, *Lcn2* was the most downregulated gene during *Cn* H99 brain infection. Reduced or deficient *Lcn2* is associated with attenuated neuroinflammation, brain injury, and neurological deficits in mice [58, 59] via suppression of pyroptosis [60], a pro-inflammatory programmed cell death different than apoptosis and necrosis and modulated by the gasdermins (GSDM) family. For example, after i.c. hemorrhage, the NOD-like receptor protein 3 (NLRP3)/Caspase-1/GSDM signaling is critical in the induction of pyroptosis, which further produces IL-1β and IL-18, as well as intracellular danger signals, resulting in the lysis and death of neurons, glial cells, or endothelial cells [60]. Since IL-1β levels and the expression of its receptor (*Il1rn*) are high in H99-infected brains, it is probable that reduction in *Lcn2* antagonizes the effects of this pro-inflammatory cytokine and serves as a protective mechanism to mitigate brain tissue damage during cryptococcosis. Similarly, several genes encoding antifungal molecules that promote innate immunity including *Lgals3* (galectin-3) and *Ptx3* (pentraxin-3) were also significantly downregulated in H99-infected brains. Galectin-3 inhibits cryptococcal growth and exerts a direct lytic effect on *Cn* extracellular vesicles [61]. While pentraxin-3 is typically expressed at 7-dpi in brains with *Cn* [62], it has shown antifungal activity (e.g., *Aspergillus fumigatus*) in *Ptx3*-deficient mice [63]. In contrast, only *Myh11* (muscle myosin heavy chain 11), *CD6* (cluster of differentiation 6) and *Or4d1* (olfactory receptor family 4 subfamily D member 1) were significantly upregulated in H99-infceted tissue. *Myh11* upregulation may be associated with blood vessel permeability due to peripheral cell infiltration to the CNS to fight the infection. *Cd6* is involved in the formation and stabilization of T-cell contacts with antigen-presenting cells, a possible response to control the infection. Moreover, *Or4d1* is involved in the olfactory signaling pathways. Although we used an i.c. mouse model of infection, the high expression of *Or4d1* indicates the important role of the olfactory region and its connection with *Cn* pathogenesis. In patients with CME, *Cn* reaches the olfactory nerve fascicles through the olfactory pathways for CSF drainage, which might serve as a source of latent cryptococcal infection [64]. In mice, rapid cryptococcal brain invasion has been observed after intranasal fungal inoculation [65]. Future work is necessary to determine the role of these DEGs in cerebral cryptococcosis and in therapeutic discovery and development.

Interestingly, brains infected with the acapsular mutant strain *cap59* also resulted in significant DEGs underscoring the importance of the capsule in the development of cerebral cryptococcosis. *Lgals3bp* (Galectin 3 Binding Protein) was the most downregulated gene in the brain of *cap59*-infected mice. *Lgals3bp* is associated with regulation of microglia responses to brain infection [66], and this might explain the extensive activation of microglia in response to the acapsular fungal infection and its early reduction from brain tissue in mice. Additionally, *Gfap* was downregulated in *cap59*-infected brains and encodes a glial fibrillary acidic protein (GFAP), a marker of astrocytes. In the human brain, astrogliosis is concentrated in regions of tissue destruction or cryptococcomas [18]. We recently showed that cryptococcal brain infection causes astrocytic reactivity or astrogliosis [27, 28], suggesting a potential role of these glial cells in combating *Cn* brain infection especially against cells lacking the capsule. We demonstrated that glial cell responses to GXM accumulation are different depending on the localization of the *Cn* infection in the brain [27]. GFAP is also critical against brain infection caused by *Staphylococcus aureus* and *Toxoplasma gondii* [67]. Further studies investigating the impact of these DEGs as well as the responses of astrocytes to GXM are warranted.

**Fig. 8:**
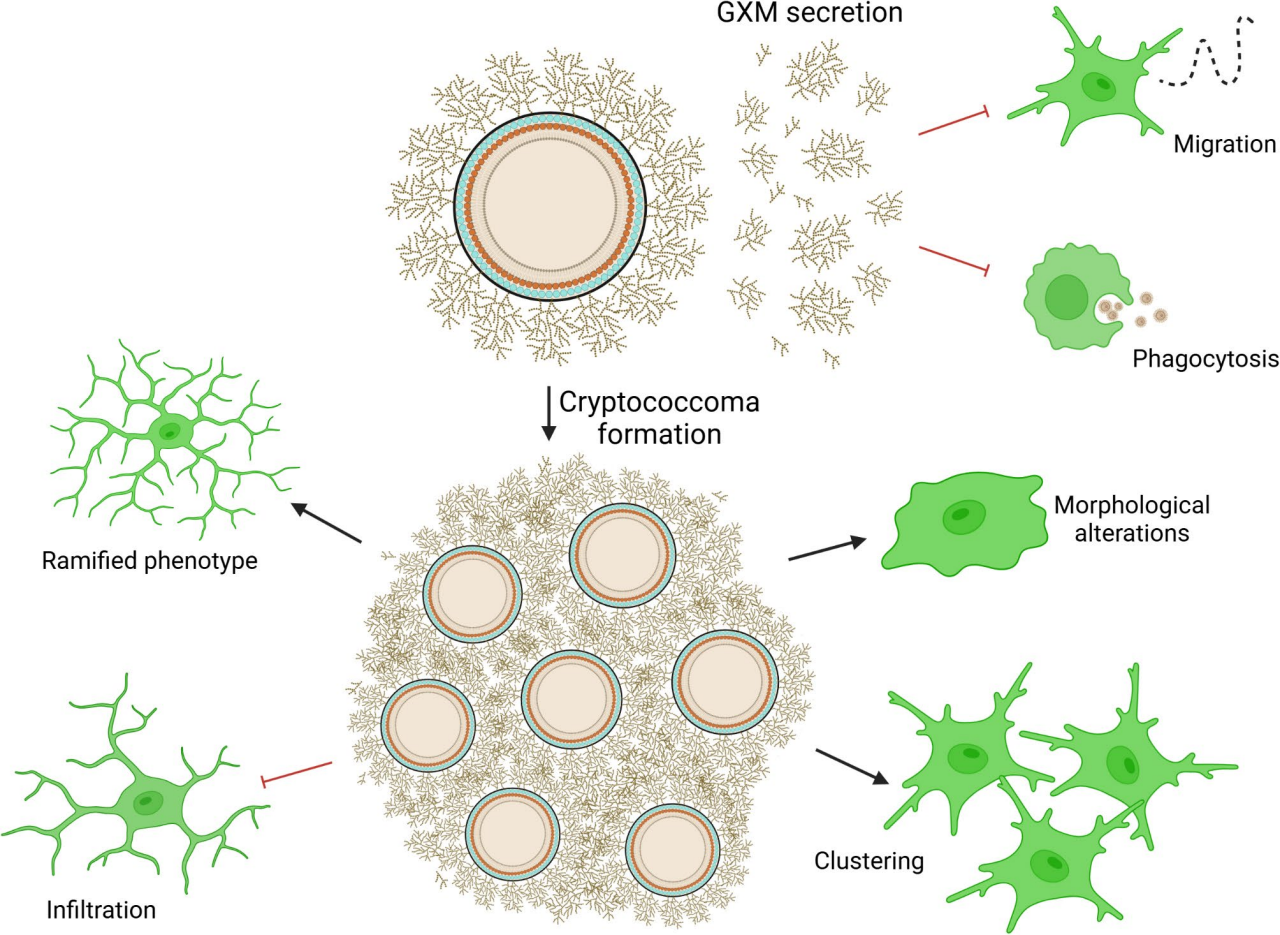
Model of *Cn* GXM-induced inhibition of microglial migration. *Cn* modulates GXM release from its capsule to potentially impede microglia from eliminating infection in vivo. * Cn* reduces microglia cell recruitment and promotes abundant cryptococcoma formation in the brain parenchyma due to increased production and release of GXM. Cryptococcomas exhibit a combination of diverse microglial morphologies that although present, make them unable to clear the infection. The diagram was created with BioRender.com by Vanessa Enriquez

We performed GO enrichment analysis in *Cn* H99-infected brain tissue after RNA-seq and identified specific DEGs mostly involved in the immune response and other homeostasis related pathways. When compared to *cap59* brain infection, mice infected with H99 demonstrated downregulation of leukocyte activation, immune cell migration, and microglial responses including phagocytosis. Our in vitro studies culturing NR-9460 microglia-like cells with H99 or *cap59* cells validated that active production of the capsule is advantageous for the fungus in inhibiting microglia chemotaxis and cryptococcal phagocytosis. Moreover, we clearly demonstrated that GXM interferes with microglial migration to the fungus (Fig. 8) and its phagocytosis. For example, GXM inhibits neutrophil migration during *Cn* infection [68] even after IL-8 production by microglia [69]. Given that GXM is polyanionic, it is plausible that this polysaccharide can cause electrostatic repulsions that prevent microglia from interacting with and eliminating the cryptococci [70]. Also, GXM-exposed microglia engulfed less cryptococci per cell than unexposed microglia. Phagocytosed cryptococci abundantly produce and secrete GXM intracellularly [71], which accumulates in the phagolysosome and results in alterations to phagolysosomal membrane and destruction of the phagocyte [71, 72]. In fact, replication of *Cn* in macrophages is accompanied by phagosomal permeabilization and accumulation of vesicles containing polysaccharide in the cytoplasm [73]. Furthermore, it is also possible that non-lytic exocytosis or vomocytosis by macrophages [74] after cryptococcal cell internalization might be driven by GXM accumulation and phagolysosomal dysregulation, although the impact of this polysaccharide on the mechanisms of this intriguing phenomenon requires more investigations.

In conclusion, we showed that microglia may not be able to control cryptococcal CNS infection and that active capsular production and release may contribute to the progression and persistence of cerebral cryptococcosis. Microglia are first responders to cryptococcal brain infection because they are found near blood capillaries [23] but they may act as an early infection reservoir [24]. Post-mortem examinations of a young individual with AIDS and cryptococcosis showed fungal cells contained in the brainstem and basal ganglia by microglial nodules with multinucleate giant cells [22], a typical manifestation in people with HIV encephalitis. Thus, it was also shown that microglia do not provide protection against *Cn* infection [24]. This is not surprising considering that a previous study revealed that *Cn* survives and replicates in human microglia [19] and activated microglia have been observed in the perivascular regions of the brain [18]. In our studies, microglia were also associated with parenchymal brain tissue, suggesting that their possible primary function in the setting of cryptococcosis involves tissue repair instead of fungal cell engulfment and elimination, with related functions in neuronal pruning [75] and CNS homeostasis [76]. Future studies are needed to evaluate this hypothesis that may be of value for the development of therapeutics and treatments to combat cerebral cryptococcosis.

## Materials and methods

**Ethics statement**. All animal studies were conducted according to the experimental practices and standards approved by the Institutional Animal Care and Use Committee (IACUC) at the University of Florida (UF; Protocol #: 202011067). The IACUC at the UF approved this study.

***Cn***. *Cn* H99 (serotype A) and *cap59* mutant strains were grown in Sabouraud dextrose broth (pH 5.6; BD Difco) for 24 h at 30 °C in an orbital shaker (New Brunswick Scientific) set at 150 rpm (nominal to early stationary phase). H99 is a wild-type *Cn* strain isolated and characterized by Dr. John Perfect [77]. The *cap59* strain was generated by the insertion of a disruptive cassette into the *cap59* gene of H99 using a biolistic DNA delivery method [29]. The *cap59* gene is required by *Cn* to produce its capsule [78].

**i.c. infection with*Cn***. CX3CR1-EGFP (The Jackson Laboratory) or C57BL/6 (Envigo) female mice (6–8 weeks old) were anesthetized using isoflurane (3–5% for induction and 1–2% maintenance; model: VetFlo Vaporizer Single Channel Anesthesia System, Kent Scientific), placed in prone position over a heating pad (model: RightTemp Jr., Kent Scientific), and prepped using standard aseptic techniques. A local anesthetic, bupivacaine or ropivacaine (0.05%; Covetrus), was administered subcutaneously in the incision. The fur on the skull was carefully shaved off and the animal was securely placed in a stereotaxic apparatus (model: 940; Kopf Instruments). Using a small hand-held microdrill (model: Ideal microdrill; Braintree Scientific), the skull was thinned until the underlying dura mater was visible and a 26 G Hamilton syringe was brought to the correct stereotaxic position and lowered until it touched the exposed dura. The craniotomy was around 1-mm in diameter and the correct brain coordinates were identified using a stereotaxic brain atlas (e.g., The Allen Mouse Brain Atlas; https://mouse.brain-map.org/static/atlas). Using the *Cn* H99 or *cap59* strains, a 1-µL suspension containing 10^4^ cryptococci in sterile saline was injected into the striatum [Stereotaxic coordinates: x (medial/lateral), -2; y (anterior/posterior), 0.2; z (dorsal/ventral), -3.5)], a region in the basal ganglia, using a 26 G Hamilton syringe connected to a pump (model: UltraMicroPump3; World Precision Instruments). Patients with HIV and CME are frequently affected in the basal ganglia where cryptococci and GXM have been shown to accumulate [22, 79–81]. Furthermore, in a retrospective study, the most observed neurological symptoms in HIV and CME patients [82] included motor and cognitive functions associated with the basal ganglia such as motor/sensory deficits and cognitive impairments (75% and 42% of patients, respectively) [83]. We injected the fungal inoculum in a 1-µL volume to avoid tissue damage or diffusion of the cryptococci to other regions of the brain. The skin incision on the dorsal head was closed with sterile nylon suture and 2–4% topical chlorhexidine solution was applied over the closed incision. After the surgery, the mice were placed in a clean recovery cage and monitored for survivability. The survival end points were inactivity, tachypnea, or loss of ≥ 25% of body weight from baseline weight. We monitored the mice twice daily for clinical signs, dehydration, and weight loss. Animals showing signs of dehydration or that lost more than 10% weight received supportive care such as 1 mL of parenteral fluid supplementation (saline) and moist chow on the cage floor was provided. In separate infections, brain tissues were excised for processing for determination of CFU numbers, histopathological studies, flow cytometry, and RNA-seq analysis.

**CFU determinations**. Brains were excised from euthanized mice and weighed at 3- and 7-dpi. The brain tissue was homogenized in 5 mL of sterile phosphate buffered saline (PBS), serially diluted, a 100 µL suspension was plated on Sabouraud dextrose agar (BD Difco) and incubated at 30 °C for 48 h. Quantification of viable yeast cells from infected animals were determined by CFU counting of two dilutions per animal (*n* = 8 per day).

**Brain histology**. CX3CR1-EGFP mice were anesthetized at 3- or 7-dpi with a ketamine (100 mg/kg; Zoetis)/xylazine (20 mg/kg; Covetrus) cocktail. A 23 G needle was inserted into the left ventricle of the heart and the right atrium was nicked to perfuse the mice with sterile saline (Baxter) followed by 4% formaldehyde (Fisher Scientific). The brains were removed and immersed in 4% paraformaldehyde overnight (O/N). Then, brains were washed thrice with sterile saline for 1 h and left O/N in 40% sucrose for cryopreservation. To embed the tissue, brains were frozen in optimal cutting temperature (OCT) compound, and 8-µm coronal tissue sections were serially cut using a cryostat (Tanner Scientific, model: TN50) and fixed onto glass slides. The slides were air-dried at room temperature (RT) prior to staining, washed twice with Tris buffered saline (TBS; Thermo Fisher), incubated in TBS (0.3% Tween 20) for 15 min, and washed again with TBS. For 3-dpi brain tissue, slides were blocked with 10% goat serum in TBS for 20 min. GXM (mAb 18B7 is an anti-cryptococcal GXM IgG_1_ generated and generously provided by Arturo Casadevall at the Johns Hopkins Bloomberg School of Public Health; 1:200 dilution; incubated O/N at 4 °C)-specific [conjugated to goat anti-mouse allophycocyanin (APC); Thermo Fisher; dilution: 1:100; incubated for 1 h at RT] immunostaining was performed to assess capsular release and distribution inside and around the cryptococcomas. Calcofluor White M2R (Thermo Fisher) was added at a 1:200 dilution for 1 h at 37 °C. For 7-dpi brain tissue, propidium iodide was used to label fungi and neurons. Samples were sealed with ProLong Gold antifade mounting reagent (Invitrogen).

**Confocal microscopy and image analysis**. The slides were visualized using a Zeiss LSM 710 inverted confocal microscope, and images captured with a Zeiss AxioCam digital camera using ZEN microscopy software (Carl Zeiss). *Z*-stack and tile stitching were used to derive composite images of the brain coronal tissue sections. Individual images were taken at 10, 20, 40, and 63X magnification. The distribution distance of microglia surrounding cryptococcomas was analyzed by measuring the radii from the microglia cell body to the edge of the cryptococcoma using NIH ImageJ software. Radar graphs plotting the radius distances were prepared in Microsoft Excel. The analysis of each microglia morphological parameters was performed using the ImageJ FIJI software. The microglia cell density and distribution were measured by placing individual dots in the cell soma or body. The NNI plugin was used to assess the nearest neighbor indices. The spacing index was determined by calculating NNI^2^ x microglial density, where the microglial density was the number of microglia divided by the ROI area [30]. Microglia clusters were counted as any nearest neighbor indices that were ≤ 12-µm. To analyze the microglia cell morphology, the cell soma and branching area were outlined to generate the soma and arborization area, respectively. Finally, the morphology index was calculated by dividing the average soma area by the average arborization area in a ROI.

**Immunolabeling of microglia and immune cells in brain tissue**. Mice were anesthetized at 7-dpi and perfused in the left ventricle with perfusate [Dulbecco’s PBS (D-PBS) + 0.5% 1000 IU/mL of heparin]. The protocol for the Multi Tissue Dissociation Kit 1 was followed. The injected hemisphere was gently sliced with a blade, inserted into a gentleMACS C tube with enzyme mix, and the tubes were placed on the gentleMACS Octo Dissociator with Heaters under the Program 37C_Multi_F. The samples were resuspended and filtered using a 70 μm filter and washed with cold D-PBS. Cells were resuspended in the ratio of D-PBS and Debris Removal Solution according to the manufacturer’s protocol. D-PBS was overlayed on the samples and the gradients were centrifuged. The top two phases were aspirated and the bottom phase was washed with D-PBS. The resulting pellet was treated with Red Blood Cell Removal Solution and neutralized with PB buffer (PBS, pH 7.2 + 0.5% bovine serum albumin (BSA) + 2 mM EDTA). Then, the pellet was washed with MACS buffer [PBS + 1% fetal bovine serum (FBS) or 0.5% BSA + 1 mM EDTA] and cells were labeled with Zombie Aqua dye for 30 min on ice in the dark. Cells were washed with PBS and resuspended in blocking buffer for 10 min on ice in the dark (MACS Buffer + 2% Normal Rat Serum + 2% Armenian Hamster Serum + 2% Normal Mouse Serum + 1% TruStain FcX Plus). Ab staining mix (prepared in blocking buffer; STable 4) was added directly to samples and incubated for 30 min on ice in the dark. Samples were washed with PBS and resuspended in 5% neutral buffered formalin (NBF) solution for 30 min on ice in the dark. Samples were washed and resuspended in FACS buffer (PBS + 3% FBS (or 1% BSA) + 10 mM EDTA + 10 mM HEPES) and analyzed or stored at 4 °C until analysis. Single stain controls were performed using BD Ultracomp Beads (Thermo Fisher). Samples were processed on a Cytek Aurora 5 cytometer and the abundance or percentage of each cell type in infected tissue was analyzed using SpectroFlo software. The relative abundance or percentage (%) of each cell type in tissue was determined from a percentage of Live CD45^+^ cells (# cell type/# CD45^+^ Live cells x 100). Uninfected brains were used as positive control.

**RNA-seq analysis**. Mice were euthanized at 7-dpi via CO_2_ inhalation, their brains were harvested, and flash-frozen in a dry ice-methylbutane slurry mix before infection sites were cut using a brain matrix. The RNA extraction was performed using the *Quick*-RNA Miniprep kit (Zymo Research) followed by an in-column DNase treatment. Isolated RNA was eluted in nuclease-free water and stored at -80 °C until RNA-seq analysis. For RNA-seq, the RNA library was prepared using the Illumina RNA-Seq Libraries Poly A kit with a normalized input of 250 ng per sample. Libraries were checked for quality (RNA integrity > 8) and quantified using the 4200 TapeStation (Agilent), before being sequenced for a minimum of 40 million reads on one S4 lane of the Illumina NovaSeq 600 instrument using 150 base paired-end sequencing at the Gene Expression and Genotyping Core Facility in the UF Interdisciplinary Center for Biotechnology Research. FastQ files, counts, and normalized counts were uploaded to the NCBI Gene Expression Omnibus (GEO) repository with accession # GSE230212. Reads acquired from the Illumina NovaSeq 6000 platform were cleaned with Trimmomatic (v0.39; [84]) for trimming adaptors and low-quality bases with a quality phred-like score < 30. Reads < 30 pair bases were excluded from RNA-seq analysis. Salmon (v1.10.2) was used to map FASTQ files and generate transcript-level counts from RNA sequencing reads using the GENCODE gene models (M33) [85]. Transcripts were mapped to genes by importing to R with the tximport package [86]. Only transcripts that mapped to a gene with known chromosomal location (i.e. no MT or unknown) and that were protein coding were retained. Differential gene expression analysis was performed using the DESeq2 R package (v1.14.1) [33]. We used a Wald test to obtain *P*-values and corrected for multiple testing using the Benjamini-Hochberg’s false discovery rate of q < 0.05. The intersections of DEGs across conditions were examined using UpSet analysis [34]. Hypergeometric tests were performed to determine if the overlap between DEGs (FDR < 0.10) and the gene sets (Gene Ontology; GO; [35, 36]), was statistically significant (Bonferroni-corrected hypergeometric *P* < 0.05). The background gene set was all protein coding genes detected in the experiment. Any gene set with < 5 genes, after filtering out genes not in the background, was excluded. GO terms that were significant with q < 0.05 were visualized using ggplot2. Metascape (v3.5; http://metascape.org; [87]) was used to further examine statistically enriched terms after filtering using accumulative hypergeometric *P*-values and enrichment factors. Metascape integrates the Pattern Gene Database (PaGenBase; http://bioinf.xmu.edu.cn/PaGenBase/) of pattern genes (e.g., specific genes, selective genes, housekeeping genes and repressed genes) of 11 model organisms identified from serial gene expression profiles of multiple physiological conditions [88].

**GXM isolation**. GXM was isolated from *Cn* H99 strain using the hexadecyltrimethyl ammonium bromide (CTAB) method as previously described [17, 89]. The *cap59* strain was used to perform a mock extraction. Briefly, 10^9^ cryptococci were inoculated into 1,000-mL Erlenmeyer flasks containing 400 mL of minimal medium composed of glucose (15 mM), MgSO4 (10 mM), KH2PO4 (29.4 mM), glycine (13 mM), and thiamine-HCl (3 µM), pH 5.5. Fungal cells were cultivated for 3 days at 30 °C with shaking and separated from culture supernatants by centrifugation at 4,000 x g (15 min, 4 °C). The supernatant fluids were collected and centrifuged again at 15,000 x g (15 min, 4 °C) to remove smaller debris. The pellets were discarded, and the resulting supernatant was concentrated approximately 20-fold using an Amicon (Millipore) ultrafiltration cell (with a cutoff of 100 kDa and a total capacity of 200 mL) with stirring and Biomax polyethersulfone ultrafiltration discs (63.5 mm). A nitrogen (N2) stream was used as the pressure gas. After the supernatant was concentrated, a thick, translucent film was observed in close association with the ultrafiltration disc and was covered by a concentrated fluid phase. The fluid phase was discarded, and the viscous layer was collected with a cell scraper for storage at RT. Fractions that were passed through the 100-kDa filtration discs were filtered through 10-kDa membranes, resulting again in film formation. We heat inactivated (100 °C for 15 min) proteases in our GXM preparation. Additionally, each preparation was treated with protease inhibitor cocktail (37 °C for 2 h). Each preparation was also tested for contamination with bacterial lipopolysaccharide (LPS) using the Limulus amoebocyte lysate (LAL) assay (Lonza). LPS was undetected in the LAL assay. We performed CFU determinations in our preparations and did not observe any culturable microbial growth. For polysaccharide quantification, a capture ELISA [90], the carbazole reaction for hexuronic acid [91], and the method for hexose detection described by Dubois et al. [92]. were used.

**Migration assay**. The microglial cell line NR-9460 (BEI Resources, NIAID, NIH) was derived using brain tissue from wild-type mice [93] and previously used by our group to study *Cn*-microglia interactions [28]. They were grown in Dulbecco’s Modified Eagle Medium (DMEM; feeding medium) supplemented with 10% FBS (R&D Systems), 5% sodium bicarbonate, 1% sodium pyruvate, and 1% penicillin-streptomycin to confluency at 37 °C in 5% CO_2_. Then, cells were trypsinized at RT, washed thrice with PBS, counted (density: 3 × 10^6^ cells/mL), and seeded into a chemotaxis chamber-slide (Ibidi) with feeding medium according to supplier’s instructions. Cells were allowed to settle O/N. Next day, 1% 4′,6-diamidino-2-phenylindole (DAPI; Molecular Probes) solution was added to the middle chamber for visibility during the migration assay, and 65-uL of feeding medium was added to the left and right chambers. For *cap59*- and H99-stimulated cells, cryptococci were added to the top right chamber port at a 10 (fungi):1 (microglia) ratio. For GXM-treated cells, the polysaccharide was isolated from *Cn* strain H99 using the hexadecyltrimethyl ammonium bromide method as previously described [89] with a few modifications [17] and 10 µg/mL of GXM instead of *Cn* were added to the top right chamber port. Then, the migration of NR-9460 cells occurred at 37 °C and 5% CO_2_ and was recorded every 10 min for 16 h using the live-imaging function (at 20X magnification) of a Zeiss LSM 710 inverted confocal microscope. The images were captured with a Zeiss AxioCam digital camera using ZEN microscopy software. Each cell was manually tracked using the Manual Tracking plugin through ImageJ FIJI software and assessed for accumulated distance with the Chemotaxis and Migration Tool software.

**Phagocytosis Assay**. Monolayers of NR-9460 cells were incubated in feeding medium supplemented with IFN-γ (100 U/mL), LPS (0.5 ng/mL), and mAb 18B7, followed by the addition of H99 or *cap59* cryptococci in a 1 (microglia):10 (yeast) ratio. The plates were incubated for 4 h at 37 °C and 5% CO_2_. To evaluate the effect of GXM on microglia-like cell phagocytosis of H99, the only modification to the above protocol was that NR-9460 cells were treated with GXM (10 µg/mL) for 2 h prior to activation with IFN-γ, LPS, and mAb 18B7. The monolayer coculture was washed thrice with PBS to remove nonadherent cells, fixed with cold methanol, Giemsa stained, and visualized with a Leica DMi8 inverted microscope, and images were captured with a Leica DFC7000 digital camera using LAS X digital imaging software. The number of internalized yeast cells per microglia was reported after counting 100 phagocytic cells per field. Internalized cells were differentiated from attached cells by the presence in a well-defined phagocytic vacuole.

**Statistical analysis**. All data were subjected to statistical analysis using Prism 10.2 (GraphPad). Differences in survival rates were analyzed by the log-rank test (Mantel-Cox). *P* values for multiple comparisons were calculated by one-way analysis of variance (ANOVA) and were adjusted by use of the Tukey’s *post-hoc* analysis. *P* values for individual comparisons were calculated using student’s or multiple *t*-test analyses. *P* values of < 0.05 were considered significant.

## Electronic supplementary material

Below is the link to the electronic supplementary material.

Supplementary SFig. 1: Representative flow cytometry dot plots show the gating strategy followed to determine the percentage of myeloid and lymphoid origin cells in brain tissue infected with *Cn* H99 or *cap59* cells at 7-dpi. Singlets were selected using FSC-A vs. FSC-H and SSC-A vs. SSC-H followed by separation by forward (FSC) and side scatter (SSC) to remove cell aggregates, small debris, or cryptococci. Live leukocytes were then selected as CD45.2-BV786^+^ and Live/Dead − using the Zombie Aqua stain. Live CD45^+^ cells were then gated to separate myeloid or lymphocytic cells. For myeloid cells, neutrophils were gated for CD11b-APC-Cy7^+^ and Ly6G-PerCP-Cy5.5^+^. The remaining cells were gated with CD45.2-BV786^+^ and CD11b-APC-Cy7^+^ for microglia, CD11b-APC-Cy7^+^ and F4/80-BV711^+^ for macrophages, CD11b-APC-Cy7^+^ and Ly6C-PE-TexasRed^+^ for monocytes, and B220-APC-A^+^ and MHC-BV650^+^ for dendritic cells. For lymphocytes, the B and T cells were gated with B220-APC-A and CD3e-PE, respectively. The T cells were further gated with CD4-BV605 and CD8a-AF700 to distinguish CD4^+^ and CD8^+^ T cells, respectively. The relative abundance or percentage (%) of each cell type in tissue was determined from a percentage of Live CD45^+^ cells (# cell type/# CD45^+^ Live cells x 100). Both uninfected and infected brains were used for the determination of consistent gates

Supplementary SFig. 2: Representative flow cytometry dot plots for immune cells in basal ganglia infected with *Cn* H99 or *cap59* cells at 7-dpi. Percentage (%) of (**A**) neutrophils, (**B**) macrophages, (**C**) monocytes, (**D**) dendritic cells, and (**E**) lymphocytes [B cells (left), CD4^+^ T cells (center), and CD8^+^ T cells] in brain tissue infected with H99 or *cap59* cells at 7-dpi. Each symbol represents an independent replicate (*n* = 3) where ≥ 10,000 events per group were measured. For **A-E**, bars and error bars denote mean values and SDs, respectively. Significance (****, *P* < 0.0001; ***, *P* < 0.001; **, *P* < 0.01) was calculated by student’s *t*-test analysis. ns denotes comparisons which are not statistically significant

Supplementary SFig. 3: Differentially expressed genes (DEGs) in C57BL/6 mice i.c. infected with *Cn*. (**A**) Principal component analysis displaying the transcriptomic clusters between naïve (black), saline-injected (purple), H99-infected (red), and *cap59*-infected (blue) brains. (**B**) Heat map comparison of samples based on hierarchical similarity. N-naïve, S-saline, H-H99, and C-*cap59*. The degree of expression is represented by blue (-1) to red (+ 3) intensity

Supplementary SFig. 4: *Cn* H99 downregulated DEGs in the mouse brain were enriched for terms related to infection and immunity. Bubble plots of GO analysis using hypergeometric tests (Bonferroni-corrected hypergeometric *P* < 0.05) of DEGs (FDR < 0.10) comparing saline-injected and H99-infected mice at 7-dpi. The background gene set was all protein coding genes detected in the experiment. Any gene set with less than 5 genes, after filtering out genes not in the background, was excluded

Supplementary SFig. 5: Downregulated DEGs after *cap59* infection of mouse brain were enriched for terms related to the immune cells and response. Bubble plots of GO analysis using hypergeometric tests (Bonferroni-corrected hypergeometric *P* < 0.05) of DEGs (FDR < 0.10) comparing saline-injected and *cap59*-infected mice at 7-dpi. The background gene set was all protein coding genes detected in the experiment. Any gene set with less than 5 genes, after filtering out genes not in the background, was excluded

Supplementary SFig. 6: Upregulated DEGs after *cap59* infection of mouse brain were enriched for terms related to neuronal parts and function. Bubble plots of GO analysis using hypergeometric tests (Bonferroni-corrected hypergeometric *P* < 0.05) of DEGs (FDR < 0.10) comparing saline-injected and *cap59*-infected mice at 7-dpi. The background gene set was all protein coding genes detected in the experiment. Any gene set with less than 5 genes, after filtering out genes not in the background, was excluded

Supplementary SFig. 7: Enriched ontology clusters across mouse brains injected with saline or infected with either H99 or *cap59*. Hierarchical clustering of top 20 overlapping enrichment ontology terms of downregulated (**A**) or upregulated (**B**) differentially expressed genes between *cap59*-infected vs. saline-injected and H99-infected vs. saline-injected brains. Statistically enriched terms were first identified, and accumulative hypergeometric *P*-values and enrichment factors were calculated and used for filtering. Significant terms were hierarchically clustered into a tree based on Kappa-statistical similarities among their gene memberships. The best *P*-value within each cluster was selected as its representative term. The heatmap cells are colored by their *P*-values; grey cells in **B** indicate the lack of enrichment for that term in the corresponding gene list. MCODE algorithm was then applied to this network to identify neighborhoods where proteins are densely connected. Each MCODE network is assigned a unique color. The analysis and visualization were performed using Metascape

Supplementary SFig. 8: Protein-protein interaction network analysis of overlapping downregulated DEGs between *cap59*-infected vs. saline-injected and H99-infected vs. saline-injected brains. All protein-protein interactions among downregulated DEGs were extracted and mapped into a PPI network. The network was then subjected to GO enrichment analysis to contextualize our findings. We then applied the MCODE algorithm to this network to identify neighborhoods where proteins are densely connected. Each MCODE network is assigned a different color. The table shows the top MCODE terms and top three annotations. Analysis and visualization were performed using Metascape

Supplementary SFig. 9: Tissue/cell-specific gene signatures of downregulated DEGs between *cap59*-infected vs. saline-injected and H99-infected vs. saline-injected brains. The heatmap cells are colored by their *P*-values; grey cells indicate the lack of enrichment for that term in the corresponding gene list. Analysis and visualization were performed using Metascape (PaGenBase tool)

Supplementary STable 1: Comparison of DEGs in the brains of saline-injected and *Cn* H99-infected C57BL/6 mice at 7-dpi

Supplementary STable 2: Comparison of DEGs in the brains of *cap59*- and *Cn* H99-infected C57BL/6 mice at 7-dpi

Supplementary STable 3: Comparison of DEGs in the brains of saline-injected and *cap59*-infected C57BL/6 mice at 7-dpi

Supplementary STable 4: Reagents used for the quantification of immune cells in vivo

## Data Availability

FastQ files, counts, and normalized counts were uploaded to the NCBI Gene Expression Omnibus (GEO) repository with accession # GSE230212.

## References

[1] Rajasingham R, Govender NP, Jordan A, Loyse A, Shroufi A, Denning DW, Meya DB, Chiller TM, Boulware DR. The global burden of HIV-associated Cryptococcal infection in adults in 2020: a modelling analysis. Lancet Infect Dis. 2022;22(12):1748–55. 36049486 10.1016/S1473-3099(22)00499-6 PMC9701154

[2] Neilson JB, Fromtling RA, Bulmer GS. Cryptococcus neoformans: size range of infectious particles from aerosolized soil. Infect Immun. 1977;17(3):634–8. 332630 10.1128/iai.17.3.634-638.1977 PMC421174

[3] Dromer F, Mathoulin-Pelissier S, Launay O, Lortholary O, French Cryptococcosis Study G. Determinants of disease presentation and outcome during cryptococcosis: the CryptoA/D study. PLoS Med. 2007;4(2):e21. 17284154 10.1371/journal.pmed.0040021 PMC1808080

[4] Lortholary O, Improvisi L, Rayhane N, Gray F, Fitting C, Cavaillon JM, Dromer F. Cytokine profiles of AIDS patients are similar to those of mice with disseminated Cryptococcus neoformans infection. Infect Immun. 1999;67(12):6314–20. 10569743 10.1128/iai.67.12.6314-6320.1999 PMC97035

[5] Chretien F, Lortholary O, Kansau I, Neuville S, Gray F, Dromer F. Pathogenesis of cerebral Cryptococcus neoformans infection after fungemia. J Infect Dis. 2002;186(4):522–30. 12195380 10.1086/341564

[6] Chang YC, Stins MF, McCaffery MJ, Miller GF, Pare DR, Dam T, Paul-Satyaseela M, Kim KS, Kwon-Chung KJ. Cryptococcal yeast cells invade the central nervous system via transcellular penetration of the blood-brain barrier. Infect Immun. 2004;72(9):4985–95. 15321990 10.1128/IAI.72.9.4985-4995.2004 PMC517459

[7] Eugenin EA, Greco JM, Frases S, Nosanchuk JD, Martinez LR. Methamphetamine alters blood brain barrier protein expression in mice, facilitating central nervous system infection by neurotropic Cryptococcus neoformans. J Infect Dis. 2013;208(4):699–704. 23532099 10.1093/infdis/jit117 PMC3719895

[8] Charlier C, Nielsen K, Daou S, Brigitte M, Chretien F, Dromer F. Evidence of a role for monocytes in dissemination and brain invasion by Cryptococcus neoformans. Infect Immun. 2009;77(1):120–7. 18936186 10.1128/IAI.01065-08 PMC2612285

[9] Santiago-Tirado FH, Onken MD, Cooper JA, Klein RS, Doering TL. Trojan horse transit contributes to Blood-Brain barrier crossing of a eukaryotic pathogen. mBio 2017, 8(1). 10.1128/mBio.02183-16 PMC5285505 28143979

[10] Butler EK, Boulware DR, Bohjanen PR, Meya DB. Long term 5-year survival of persons with Cryptococcal meningitis or asymptomatic subclinical antigenemia in Uganda. PLoS ONE. 2012;7(12):e51291. 23251485 10.1371/journal.pone.0051291 PMC3519582

[11] Fromtling RA, Shadomy HJ, Jacobson ES. Decreased virulence in stable, acapsular mutants of cryptococcus neoformans. Mycopathologia. 1982;79(1):23–9. 6750405 10.1007/BF00636177

[12] Cherniak R, Sundstrom JB. Polysaccharide antigens of the capsule of Cryptococcus neoformans. Infect Immun. 1994;62(5):1507–12. 8168912 10.1128/iai.62.5.1507-1512.1994 PMC186341

[13] Goldman DL, Lee SC, Casadevall A. Tissue localization of Cryptococcus neoformans glucuronoxylomannan in the presence and absence of specific antibody. Infect Immun. 1995;63(9):3448–53. 7642276 10.1128/iai.63.9.3448-3453.1995 PMC173475

[14] Vecchiarelli A. Immunoregulation by capsular components of Cryptococcus neoformans. Med Mycol. 2000;38(6):407–17. 11204878 10.1080/mmy.38.6.407.417

[15] Lee SC, Casadevall A, Dickson DW. Immunohistochemical localization of capsular polysaccharide antigen in the central nervous system cells in Cryptococcal meningoencephalitis. Am J Pathol. 1996;148(4):1267–74. 8644867 PMC1861512

[16] Pettoello-Mantovani M, Casadevall A, Smarnworawong P, Goldstein H. Enhancement of HIV type 1 infectivity in vitro by capsular polysaccharide of Cryptococcus neoformans and Haemophilus influenzae. AIDS Res Hum Retroviruses. 1994;10(9):1079–87. 7826695 10.1089/aid.1994.10.1079

[17] Lee HH, Carmichael DJ, Ribeiro V, Parisi DN, Munzen ME, Charles-Nino CL, Hamed MF, Kaur E, Mishra A, Patel J, et al. Glucuronoxylomannan intranasal challenge prior to Cryptococcus neoformans pulmonary infection enhances cerebral cryptococcosis in rodents. PLoS Pathog. 2023;19(4):e1010941. 37115795 10.1371/journal.ppat.1010941 PMC10171644

[18] Lee SC, Dickson DW, Casadevall A. Pathology of Cryptococcal meningoencephalitis: analysis of 27 patients with pathogenetic implications. Hum Pathol. 1996;27(8):839–47. 8760020 10.1016/s0046-8177(96)90459-1

[19] Lee SC, Kress Y, Zhao ML, Dickson DW, Casadevall A. Cryptococcus neoformans survive and replicate in human microglia. Lab Invest. 1995;73(6):871–9. 8558850

[20] Buchanan KL, Doyle HA. Requirement for CD4(+) T lymphocytes in host resistance against Cryptococcus neoformans in the central nervous system of immunized mice. Infect Immun. 2000;68(2):456–62. 10639404 10.1128/iai.68.2.456-462.2000 PMC97163

[21] Goldman D, Song X, Kitai R, Casadevall A, Zhao ML, Lee SC. Cryptococcus neoformans induces macrophage inflammatory protein 1alpha (MIP-1alpha) and MIP-1beta in human microglia: role of specific antibody and soluble capsular polysaccharide. Infect Immun. 2001;69(3):1808–15. 11179358 10.1128/IAI.69.3.1808-1815.2001 PMC98087

[22] Edelman M, Birkenhauer MC, Steinberg JJ, Dickson DW, Casadevall A, Lee SC. Microglial nodule encephalitis: limited CNS infection despite disseminated systemic cryptococcosis. Clin Neuropathol. 1996;15(1):30–3. 8998854

[23] Francis VI, Liddle C, Camacho E, Kulkarni M, Junior SRS, Harvey JA, Ballou ER, Thomson DD, Brown GD, Hardwick JM, et al. Cryptococcus neoformans rapidly invades the murine brain by sequential breaching of airway and endothelial tissues barriers, followed by engulfment by microglia. mBio. 2024;15(4):e0307823. 38511961 10.1128/mbio.03078-23 PMC11005363

[24] Mohamed SH, Fu MS, Hain S, Alselami A, Vanhoffelen E, Li Y, Bojang E, Lukande R, Ballou ER, May RC, et al. Microglia are not protective against Cryptococcal meningitis. Nat Commun. 2023;14(1):7202. 37938547 10.1038/s41467-023-43061-0 PMC10632471

[25] Aslanyan L, Sanchez DA, Valdebenito S, Eugenin EA, Ramos RL, Martinez LR. The crucial role of biofilms in Cryptococcus neoformans survival within macrophages and colonization of the central nervous system. J Fungi (Basel) 2017, 3(1). 10.3390/jof3010010 PMC5715963 29371529

[26] Koutsouras GW, Ramos RL, Martinez LR. Role of microglia in fungal infections of the central nervous system. Virulence. 2017;8(6):705–18. 27858519 10.1080/21505594.2016.1261789 PMC5626199

[27] Hamed MF, Enriquez V, Munzen ME, Charles-Nino CL, Mihu MR, Khoshbouei H, Alvina K, Martinez LR. Clinical and pathological characterization of central nervous system cryptococcosis in an experimental mouse model of stereotaxic intracerebral infection. PLoS Negl Trop Dis. 2023;17(1):e0011068. 36656900 10.1371/journal.pntd.0011068 PMC9888703

[28] Hamed MF, Araujo GRS, Munzen ME, Reguera-Gomez M, Epstein C, Lee HH, Frases S, Martinez LR. Phospholipase B is critical for Cryptococcus neoformans survival in the central nervous system. mBio. 2023;14(2):e0264022. 36786559 10.1128/mbio.02640-22 PMC10127605

[29] Nelson RT, Pryor BA, Lodge JK. Sequence length required for homologous recombination in Cryptococcus neoformans. Fungal Genet Biol. 2003;38(1):1–9. 12553931 10.1016/s1087-1845(02)00510-8

[30] Gonzalez Ibanez F, Picard K, Bordeleau M, Sharma K, Bisht K, Tremblay ME. Immunofluorescence staining using IBA1 and TMEM119 for microglial density, morphology and peripheral myeloid cell infiltration analysis in mouse brain. J Vis Exp 2019(152). 10.3791/60510 31710033

[31] Tomaszewski WH, Waibl-Polania J, Miggelbrink AM, Chakraborty MA, Fecci PE, Sampson JH, Gunn MD. Broad immunophenotyping of the murine brain tumor microenvironment. J Immunol Methods. 2021;499:113158. 34597618 10.1016/j.jim.2021.113158 PMC8608727

[32] Davoust N, Vuaillat C, Cavillon G, Domenget C, Hatterer E, Bernard A, Dumontel C, Jurdic P, Malcus C, Confavreux C, et al. Bone marrow CD34+/B220 + progenitors target the inflamed brain and display in vitro differentiation potential toward microglia. FASEB J. 2006;20(12):2081–92. 17012260 10.1096/fj.05-5593com

[33] Love MI, Huber W, Anders S. Moderated Estimation of fold change and dispersion for RNA-seq data with DESeq2. Genome Biol. 2014;15(12):550. 25516281 10.1186/s13059-014-0550-8 PMC4302049

[34] Lex A, Gehlenborg N, Strobelt H, Vuillemot R, Pfister H. UpSet: visualization of intersecting sets. IEEE Trans Vis Comput Graph. 2014;20(12):1983–92. 26356912 10.1109/TVCG.2014.2346248 PMC4720993

[35] Ashburner M, Ball CA, Blake JA, Botstein D, Butler H, Cherry JM, Davis AP, Dolinski K, Dwight SS, Eppig JT, et al. Gene ontology: tool for the unification of biology. The gene ontology consortium. Nat Genet. 2000;25(1):25–9. 10802651 10.1038/75556 PMC3037419

[36] Harris MA, Clark J, Ireland A, Lomax J, Ashburner M, Foulger R, Eilbeck K, Lewis S, Marshall B, Mungall C, et al. The gene ontology (GO) database and informatics resource. Nucleic Acids Res. 2004;32(Database issue):D258–261. 14681407 10.1093/nar/gkh036 PMC308770

[37] Chiapello LS, Aoki MP, Rubinstein HR, Masih DT. Apoptosis induction by glucuronoxylomannan of Cryptococcus neoformans. Med Mycol. 2003;41(4):347–53. 12964728 10.1080/1369378031000137260

[38] Pericolini E, Cenci E, Monari C, De Jesus M, Bistoni F, Casadevall A, Vecchiarelli A. Cryptococcus neoformans capsular polysaccharide component Galactoxylomannan induces apoptosis of human T-cells through activation of caspase-8. Cell Microbiol. 2006;8(2):267–75. 16441437 10.1111/j.1462-5822.2005.00619.x

[39] Welser-Alves JV, Milner R. Microglia are the major source of TNF-alpha and TGF-beta1 in postnatal glial cultures; regulation by cytokines, lipopolysaccharide, and vitronectin. Neurochem Int. 2013;63(1):47–53. 23619393 10.1016/j.neuint.2013.04.007 PMC3819935

[40] Barluzzi R, Brozzetti A, Delfino D, Bistoni F, Blasi E. Role of the capsule in microglial cell-Cryptococcus neoformans interaction: impairment of antifungal activity but not of secretory functions. Med Mycol. 1998;36(4):189–97. 9776834

[41] De Leon-Rodriguez CM, Fu MS, Corbali MO, Cordero RJB, Casadevall A. The Capsule of Cryptococcus neoformans Modulates Phagosomal pH through Its Acid-Base Properties. mSphere. 2018;3(5). 10.1128/mSphere.00437-18 PMC6200979 30355667

[42] Gensel JC, Zhang B. Macrophage activation and its role in repair and pathology after spinal cord injury. Brain Res. 2015;1619:1–11. 25578260 10.1016/j.brainres.2014.12.045

[43] Penkowa M, Giralt M, Lago N, Camats J, Carrasco J, Hernandez J, Molinero A, Campbell IL, Hidalgo J. Astrocyte-targeted expression of IL-6 protects the CNS against a focal brain injury. Exp Neurol. 2003;181(2):130–48. 12781987 10.1016/s0014-4886(02)00051-1

[44] Blasi E, Barluzzi R, Mazzolla R, Mosci P, Bistoni F. Experimental model of intracerebral infection with Cryptococcus neoformans: roles of phagocytes and opsonization. Infect Immun. 1992;60(9):3682–8. 1500177 10.1128/iai.60.9.3682-3688.1992 PMC257377

[45] Davis BM, Salinas-Navarro M, Cordeiro MF, Moons L, De Groef L. Characterizing microglia activation: a Spatial statistics approach to maximize information extraction. Sci Rep. 2017;7(1):1576. 28484229 10.1038/s41598-017-01747-8 PMC5431479

[46] Chen LC, Goldman DL, Doering TL, Pirofski L, Casadevall A. Antibody response to Cryptococcus neoformans proteins in rodents and humans. Infect Immun. 1999;67(5):2218–24. 10225877 10.1128/iai.67.5.2218-2224.1999 PMC115960

[47] Gupta S, Ellis M, Cesario T, Ruhling M, Vayuvegula B. Disseminated Cryptococcal infection in a patient with hypogammaglobulinemia and normal T cell functions. Am J Med. 1987;82(1):129–31. 3492141 10.1016/0002-9343(87)90388-3

[48] Subramaniam K, Metzger B, Hanau LH, Guh A, Rucker L, Badri S, Pirofski LA. IgM(+) memory B cell expression predicts HIV-associated cryptococcosis status. J Infect Dis. 2009;200(2):244–51. 19527168 10.1086/599318 PMC2805277

[49] Suzuki SML, Morelli F, Negri M, Bonfim-Mendonca P, Kioshima ES, Salci T, Voidaleski MF, Vicente VA, Svidzinski T. FATAL Cryptococcal meningitis in a child with hyper-immunoglobulin M syndrome, with an emphasis on the agent. J Mycol Med. 2019;29(3):273–7. 31409527 10.1016/j.mycmed.2019.07.002

[50] Michael BD, Bricio-Moreno L, Sorensen EW, Miyabe Y, Lian J, Solomon T, Kurt-Jones EA, Luster AD. Astrocyte- and Neuron-Derived CXCL1 drives neutrophil transmigration and Blood-Brain barrier permeability in viral encephalitis. Cell Rep. 2020;32(11):108150. 32937134 10.1016/j.celrep.2020.108150 PMC7548103

[51] Zhou J, Stohlman SA, Hinton DR, Marten NW. Neutrophils promote mononuclear cell infiltration during viral-induced encephalitis. J Immunol. 2003;170(6):3331–6. 12626593 10.4049/jimmunol.170.6.3331

[52] Zhang M, Sun D, Liu G, Wu H, Zhou H, Shi M. Real-time in vivo imaging reveals the ability of neutrophils to remove Cryptococcus neoformans directly from the brain vasculature. J Leukoc Biol. 2016;99(3):467–73. 26428677 10.1189/jlb.4AB0715-281R PMC6608047

[53] Sun D, Zhang M, Sun P, Liu G, Strickland AB, Chen Y, Fu Y, Yosri M, Shi M. VCAM1/VLA4 interaction mediates Ly6Clow monocyte recruitment to the brain in a TNFR signaling dependent manner during fungal infection. PLoS Pathog. 2020;16(2):e1008361. 32101593 10.1371/journal.ppat.1008361 PMC7062284

[54] Xu J, Hissong R, Bareis R, Creech A, Goughenour KD, Freeman CM, Olszewski MA. Batf3-dependent orchestration of the robust Th1 responses and fungal control during Cryptococcal infection, the role of cDC1. mBio. 2024;15(3):e0285323. 38349130 10.1128/mbio.02853-23 PMC10936214

[55] Gyoneva S, Ransohoff RM. Inflammatory reaction after traumatic brain injury: therapeutic potential of targeting cell-cell communication by chemokines. Trends Pharmacol Sci. 2015;36(7):471–80. 25979813 10.1016/j.tips.2015.04.003 PMC4485943

[56] Xu J, Ganguly A, Zhao J, Ivey M, Lopez R, Osterholzer JJ, Cho CS, Olszewski MA. CCR2 signaling promotes brain infiltration of inflammatory monocytes and contributes to neuropathology during Cryptococcal meningoencephalitis. mBio. 2021;12(4):e0107621. 34311579 10.1128/mBio.01076-21 PMC8406332

[57] Supasorn O, Sringkarin N, Srimanote P, Angkasekwinai P. Matrix metalloproteinases contribute to the regulation of chemokine expression and pulmonary inflammation in Cryptococcus infection. Clin Exp Immunol. 2016;183(3):431–40. 26445891 10.1111/cei.12725 PMC4750603

[58] Jin M, Kim JH, Jang E, Lee YM, Soo Han H, Woo DK, Park DH, Kook H, Suk K. Lipocalin-2 deficiency attenuates neuroinflammation and brain injury after transient middle cerebral artery occlusion in mice. J Cereb Blood Flow Metab. 2014;34(8):1306–14. 24780901 10.1038/jcbfm.2014.83 PMC4126090

[59] Ni W, Zheng M, Xi G, Keep RF, Hua Y. Role of lipocalin-2 in brain injury after intracerebral hemorrhage. J Cereb Blood Flow Metab. 2015;35(9):1454–61. 25853903 10.1038/jcbfm.2015.52 PMC4640334

[60] Zhao Y, Xiao Q, Sun T, Yu H, Luo M. Knockdown of LCN2 attenuates brain injury after intracerebral hemorrhage via suppressing pyroptosis. Neuropsychiatr Dis Treat. 2024;20:83–99. 38249526 10.2147/NDT.S440065 PMC10800110

[61] Almeida F, Wolf JM, da Silva TA, DeLeon-Rodriguez CM, Rezende CP, Pessoni AM, Fernandes FF, Silva-Rocha R, Martinez R, Rodrigues ML, et al. Galectin-3 impacts Cryptococcus neoformans infection through direct antifungal effects. Nat Commun. 2017;8(1):1968. 29213074 10.1038/s41467-017-02126-7 PMC5719036

[62] Polentarutti N, Bottazzi B, Di Santo E, Blasi E, Agnello D, Ghezzi P, Introna M, Bartfai T, Richards G, Mantovani A. Inducible expression of the long pentraxin PTX3 in the central nervous system. J Neuroimmunol. 2000;106(1–2):87–94. 10814786 10.1016/s0165-5728(00)00214-9

[63] Garlanda C, Hirsch E, Bozza S, Salustri A, De Acetis M, Nota R, Maccagno A, Riva F, Bottazzi B, Peri G, et al. Non-redundant role of the long pentraxin PTX3 in anti-fungal innate immune response. Nature. 2002;420(6912):182–6. 12432394 10.1038/nature01195

[64] Lima C, Vital JP. Olfactory pathways in three patients with Cryptococcal meningitis and acquired immune deficiency syndrome. J Neurol Sci. 1994;123(1–2):195–9. 8064314 10.1016/0022-510x(94)90223-2

[65] Coelho C, Camacho E, Salas A, Alanio A, Casadevall A. Intranasal Inoculation of Cryptococcus neoformans in Mice Produces Nasal Infection with Rapid Brain Dissemination. mSphere. 2019;4(4). 10.1128/mSphere.00483-19 PMC6686232 31391283

[66] Arutyunov A, Durán-Laforet V, Ai S, Ferrari L, Murphy R, Schafer DP, Klein RS. West nile Virus-Induced expression of senescent gene Lgals3bp regulates microglial phenotype within cerebral cortex. Biomolecules. 2024;14:808. 39062523 10.3390/biom14070808 PMC11274721

[67] Stenzel W, Soltek S, Schluter D, Deckert M. The intermediate filament GFAP is important for the control of experimental murine Staphylococcus aureus-induced brain abscess and Toxoplasma encephalitis. J Neuropathol Exp Neurol. 2004;63(6):631–40. 15217091 10.1093/jnen/63.6.631

[68] Dong ZM, Murphy JW. Mobility of human neutrophils in response to Cryptococcus neoformans cells, culture filtrate antigen, and individual components of the antigen. Infect Immun. 1993;61(12):5067–77. 8225584 10.1128/iai.61.12.5067-5077.1993 PMC281285

[69] Lipovsky MM, Gekker G, Hu S, Ehrlich LC, Hoepelman AI, Peterson PK. Cryptococcal glucuronoxylomannan induces Interleukin (IL)-8 production by human microglia but inhibits neutrophil migration toward IL-8. J Infect Dis. 1998;177(1):260–3. 9419203 10.1086/517368

[70] Nosanchuk JD, Casadevall A. Cellular charge of Cryptococcus neoformans: contributions from the capsular polysaccharide, melanin, and monoclonal antibody binding. Infect Immun. 1997;65(5):1836–41. 9125569 10.1128/iai.65.5.1836-1841.1997 PMC175227

[71] Goldman DL, Lee SC, Mednick AJ, Montella L, Casadevall A. Persistent Cryptococcus neoformans pulmonary infection in the rat is associated with intracellular parasitism, decreased inducible nitric oxide synthase expression, and altered antibody responsiveness to Cryptococcal polysaccharide. Infect Immun. 2000;68(2):832–8. 10639453 10.1128/iai.68.2.832-838.2000 PMC97212

[72] Feldmesser M, Kress Y, Novikoff P, Casadevall A. Cryptococcus neoformans is a facultative intracellular pathogen in murine pulmonary infection. Infect Immun. 2000;68(7):4225–37. 10858240 10.1128/iai.68.7.4225-4237.2000 PMC101732

[73] Tucker SC, Casadevall A. Replication of Cryptococcus neoformans in macrophages is accompanied by phagosomal permeabilization and accumulation of vesicles containing polysaccharide in the cytoplasm. Proc Natl Acad Sci U S A. 2002;99(5):3165–70. 11880650 10.1073/pnas.052702799 PMC122490

[74] Alvarez M, Casadevall A. Phagosome extrusion and host-cell survival after Cryptococcus neoformans phagocytosis by macrophages. Curr Biol. 2006;16(21):2161–5. 17084702 10.1016/j.cub.2006.09.061

[75] Geloso MC, D’Ambrosi N. Microglial pruning: relevance for synaptic dysfunction in multiple sclerosis and related experimental models. Cells 2021, 10(3). 10.3390/cells10030686 PMC8003660 33804596

[76] Li Q, Barres BA. Microglia and macrophages in brain homeostasis and disease. Nat Rev Immunol. 2018;18(4):225–42. 29151590 10.1038/nri.2017.125

[77] Perfect JR, Lang SD, Durack DT. Chronic Cryptococcal meningitis: a new experimental model in rabbits. Am J Pathol. 1980;101(1):177–94. 7004196 PMC1903580

[78] Chang YC, Kwon-Chung KJ. Complementation of a capsule-deficient mutation of Cryptococcus neoformans restores its virulence. Mol Cell Biol. 1994;14(7):4912–9. 8007987 10.1128/mcb.14.7.4912-4919.1994 PMC358863

[79] Klock C, Cerski M, Goldani LZ. Histopathological aspects of neurocryptococcosis in HIV-infected patients: autopsy report of 45 patients. Int J Surg Pathol. 2009;17(6):444–8. 18611927 10.1177/1066896908320550

[80] Kondo R, Sugita Y, Arakawa K, Nakashima S, Umeno Y, Todoroki K, Yoshida T, Takase Y, Kage M, Oshima K, et al. Neurogenic pulmonary edema following Cryptococcal meningoencephalitis associated with HIV infection. Neuropathology. 2015;35(4):343–7. 25955768 10.1111/neup.12193

[81] Miszkiel KA, Hall-Craggs MA, Miller RF, Kendall BE, Wilkinson ID, Paley MN, Harrison MJ. The spectrum of MRI findings in CNS cryptococcosis in AIDS. Clin Radiol. 1996;51(12):842–50. 8972648 10.1016/s0009-9260(96)80080-8

[82] Chen H, Lin F, Liu S, Da Y, Guo D. Neurological manifestations, laboratory and neuroimaging features in HIV-infected patients. Neurosciences (Riyadh). 2017;22(4):311–5. 29057859 10.17712/nsj.2017.4.20160606 PMC5946383

[83] Leisman G, Melillo R. The basal ganglia: motor and cognitive relationships in a clinical neurobehavioral context. Rev Neurosci. 2013;24(1):9–25. 23241666 10.1515/revneuro-2012-0067

[84] Bolger AM, Lohse M, Usadel B. Trimmomatic: a flexible trimmer for illumina sequence data. Bioinformatics. 2014;30(15):2114–20. 24695404 10.1093/bioinformatics/btu170 PMC4103590

[85] Patro R, Duggal G, Love MI, Irizarry RA, Kingsford C. Salmon provides fast and bias-aware quantification of transcript expression. Nat Methods. 2017;14(4):417–9. 28263959 10.1038/nmeth.4197 PMC5600148

[86] Soneson C, Love MI, Robinson MD. Differential analyses for RNA-seq: transcript-level estimates improve gene-level inferences. F1000Res. 2015;4:1521. 10.12688/f1000research.7563.1 PMC4712774 26925227

[87] Zhou Y, Zhou B, Pache L, Chang M, Khodabakhshi AH, Tanaseichuk O, Benner C, Chanda SK. Metascape provides a biologist-oriented resource for the analysis of systems-level datasets. Nat Commun. 2019;10(1):1523. 30944313 10.1038/s41467-019-09234-6 PMC6447622

[88] Pan JB, Hu SC, Shi D, Cai MC, Li YB, Zou Q, Ji ZL. PaGenBase: a pattern gene database for the global and dynamic Understanding of gene function. PLoS ONE. 2013;8(12):e80747. 24312499 10.1371/journal.pone.0080747 PMC3846610

[89] Nimrichter L, Frases S, Cinelli LP, Viana NB, Nakouzi A, Travassos LR, Casadevall A, Rodrigues ML. Self-aggregation of Cryptococcus neoformans capsular glucuronoxylomannan is dependent on divalent cations. Eukaryot Cell. 2007;6(8):1400–10. 17573547 10.1128/EC.00122-07 PMC1951138

[90] Casadevall A, Mukherjee J, Scharff MD. Monoclonal antibody based ELISAs for Cryptococcal polysaccharide. J Immunol Methods. 1992;154(1):27–35. 1401941 10.1016/0022-1759(92)90209-c

[91] Dische Z. A specific color reaction for glucuronic acid. J Biol Chem. 1947;171(2):725–30. 20272112

[92] Dubois M, Gilles K, Hamilton JK, Rebers PA, Smith F. A colorimetric method for the determination of sugars. Nature. 1951;168(4265):167. 14875032 10.1038/168167a0

[93] Blasi E, Mathieson BJ, Varesio L, Cleveland JL, Borchert PA, Rapp UR. Selective immortalization of murine macrophages from fresh bone marrow by a Raf/myc Recombinant murine retrovirus. Nature. 1985;318(6047):667–70. 4079980 10.1038/318667a0

