# Supplementary figures and images for: Active Cryptococcus neoformans glucuronoxylomannan production prevents elimination of cryptococcal CNS infection in vivo
Source: J Neuroinflammation. 2025 Mar 4;22:61. doi: 10.1186/s12974-025-03384-9 (PMC11877788; doi:10.1186/s12974-025-03384-9)

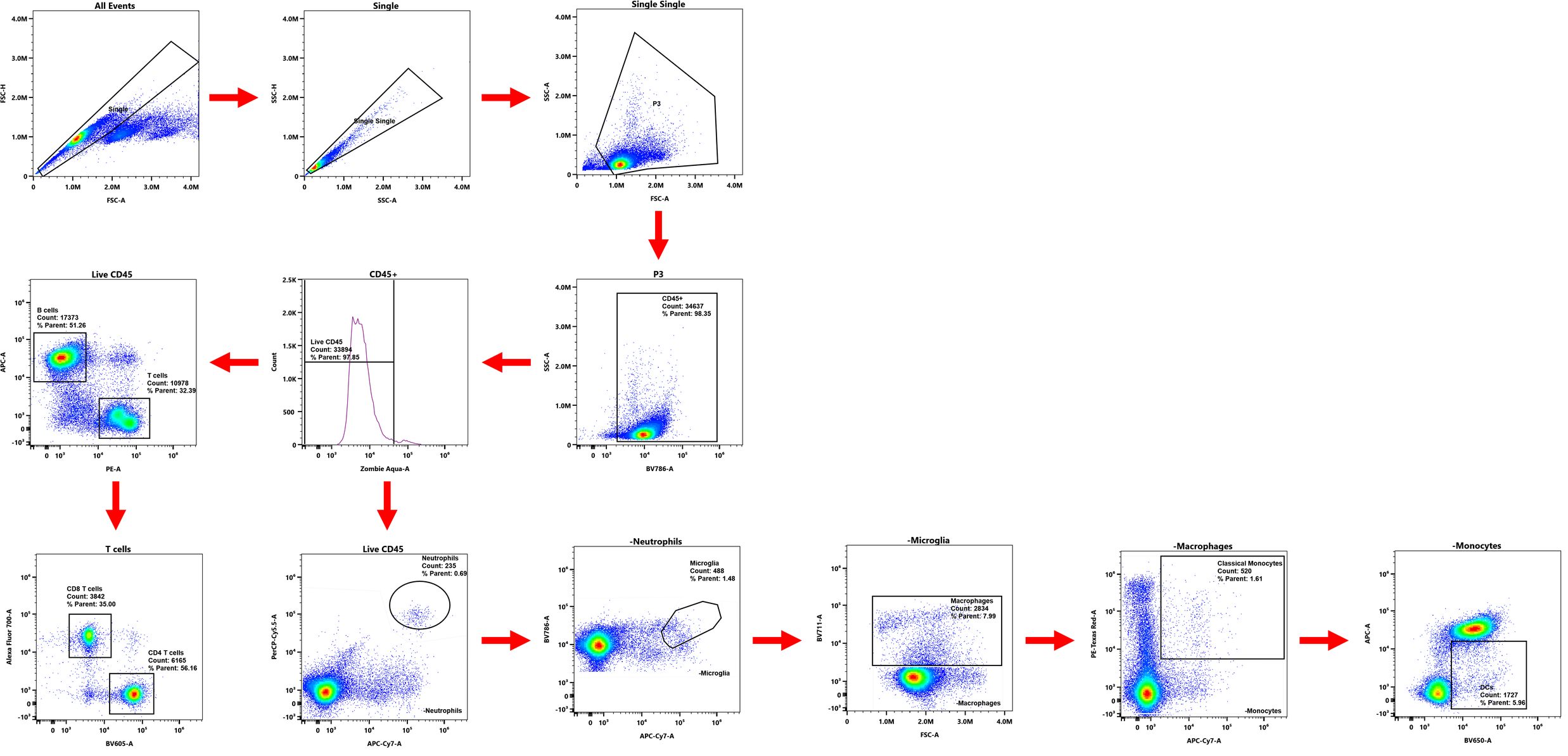

Supplement: Supplementary file 1 — Supplementary SFig. 1: Representative flow cytometry dot plots show the gating strategy followed to determine the percentage of myeloid and lymphoid origin cells in brain tissue infected with Cn H99 or cap59 cells at 7-dpi. Singlets were selected using FSC-A vs. FSC-H and SSC-A vs. SSC-H followed by separation by forward (FSC) and side scatter (SSC) to remove cell aggregates, small debris, or cryptococci. Live leukocytes were then selected as CD45.2-BV786+ and Live/Dead − using the Zombie Aqua stain. Live CD45+ cells were then gated to separate myeloid or lymphocytic cells. For myeloid cells, neutrophils were gated for CD11b-APC-Cy7+ and Ly6G-PerCP-Cy5.5+. The remaining cells were gated with CD45.2-BV786+ and CD11b-APC-Cy7+ for microglia, CD11b-APC-Cy7+ and F4/80-BV711+ for macrophages, CD11b-APC-Cy7+ and Ly6C-PE-TexasRed+ for monocytes, and B220-APC-A+ and MHC-BV650+ for dendritic cells. For lymphocytes, the B and T cells were gated with B220-APC-A and CD3e-PE, respectively. The T cells were further gated with CD4-BV605 and CD8a-AF700 to distinguish CD4+ and CD8+ T cells, respectively. The relative abundance or percentage (%) of each cell type in tissue was determined from a percentage of Live CD45+ cells (# cell type/# CD45+ Live cells x 100). Both uninfected and infected brains were used for the determination of consistent gates [file 12974_2025_3384_MOESM1_ESM.png]

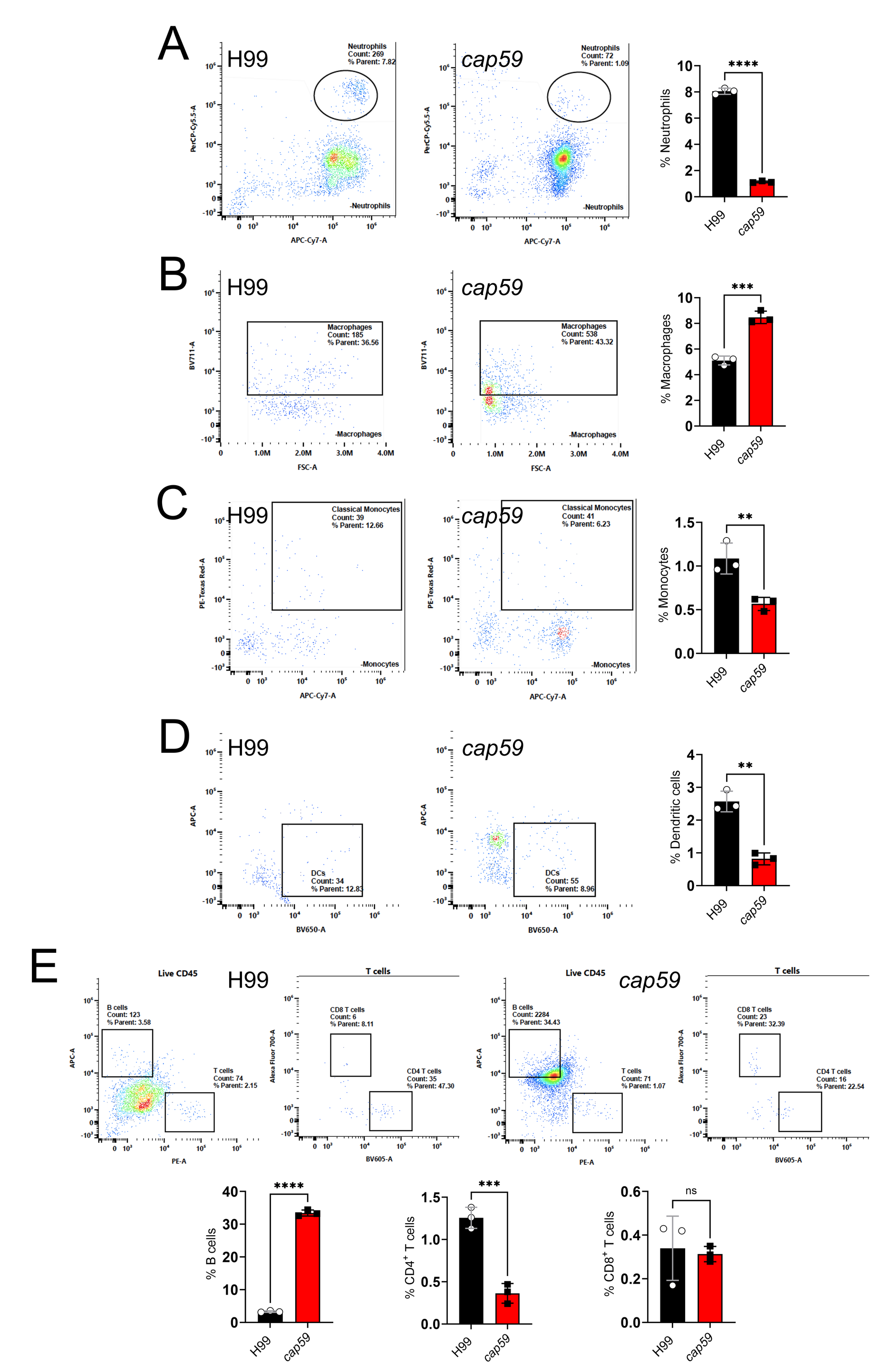

Supplement: Supplementary file 2 — Supplementary SFig. 2: Representative flow cytometry dot plots for immune cells in basal ganglia infected with Cn H99 or cap59 cells at 7-dpi. Percentage (%) of (A) neutrophils, (B) macrophages, (C) monocytes, (D) dendritic cells, and (E) lymphocytes [B cells (left), CD4+ T cells (center), and CD8+ T cells] in brain tissue infected with H99 or cap59 cells at 7-dpi. Each symbol represents an independent replicate (n = 3) where ≥ 10,000 events per group were measured. For A-E, bars and error bars denote mean values and SDs, respectively. Significance (****, P < 0.0001; ***, P < 0.001; **, P < 0.01) was calculated by student’s t-test analysis. ns denotes comparisons which are not statistically significant [file 12974_2025_3384_MOESM2_ESM.png]

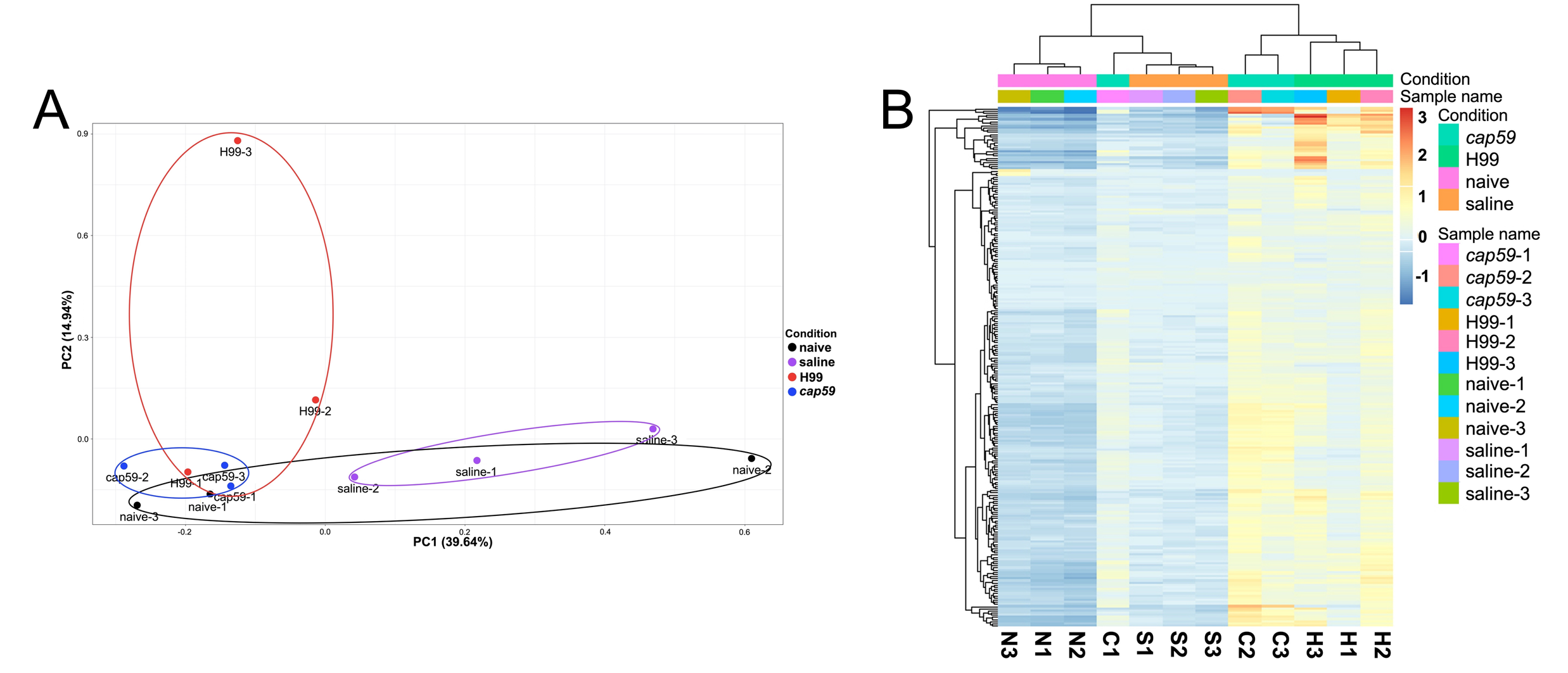

Supplement: Supplementary file 3 — Supplementary SFig. 3: Differentially expressed genes (DEGs) in C57BL/6 mice i.c. infected with Cn. (A) Principal component analysis displaying the transcriptomic clusters between naïve (black), saline-injected (purple), H99-infected (red), and cap59-infected (blue) brains. (B) Heat map comparison of samples based on hierarchical similarity. N-naïve, S-saline, H-H99, and C-cap59. The degree of expression is represented by blue (-1) to red (+ 3) intensity [file 12974_2025_3384_MOESM3_ESM.png]

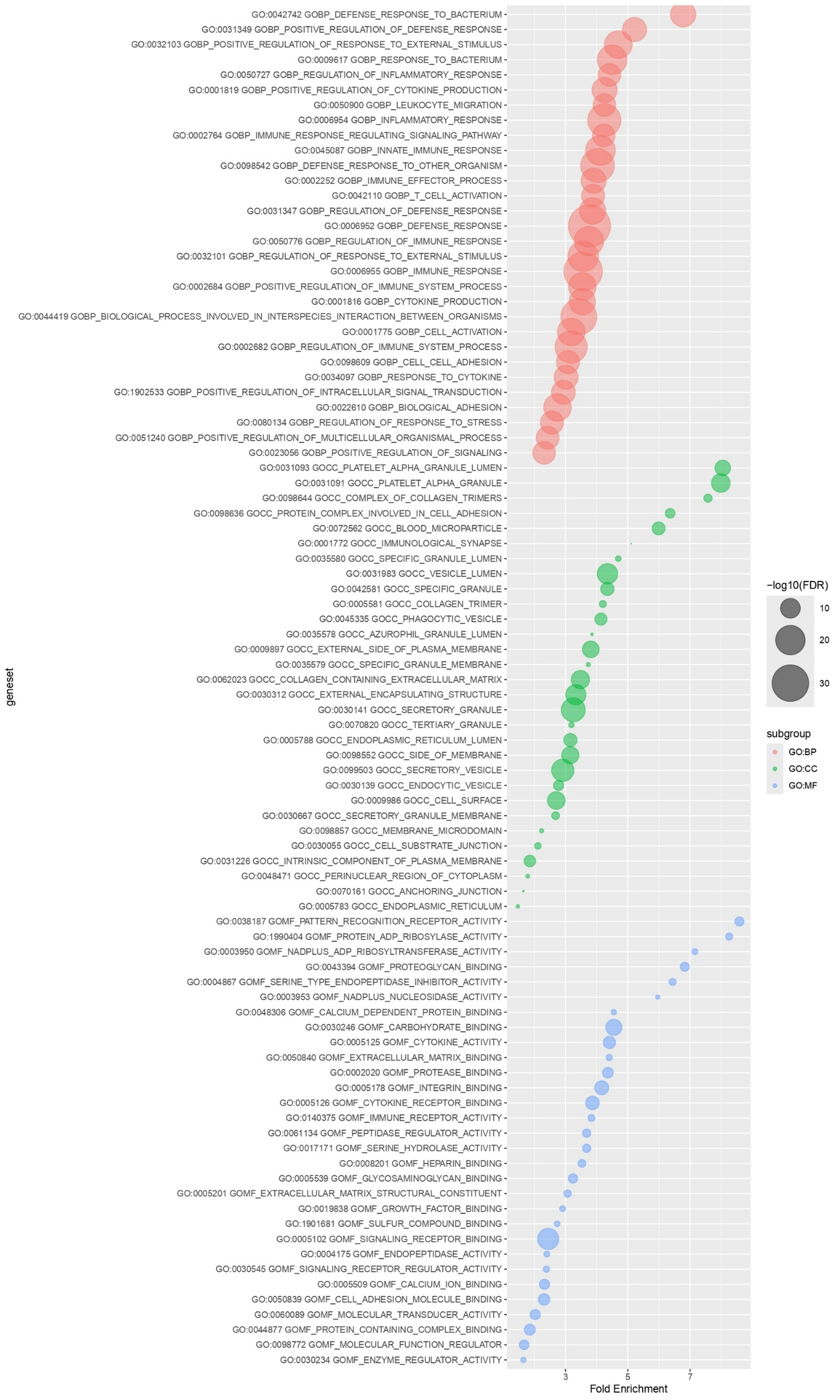

Supplement: Supplementary file 4 — Supplementary SFig. 4: Cn H99 downregulated DEGs in the mouse brain were enriched for terms related to infection and immunity. Bubble plots of GO analysis using hypergeometric tests (Bonferroni-corrected hypergeometric P < 0.05) of DEGs (FDR < 0.10) comparing saline-injected and H99-infected mice at 7-dpi. The background gene set was all protein coding genes detected in the experiment. Any gene set with less than 5 genes, after filtering out genes not in the background, was excluded [file 12974_2025_3384_MOESM4_ESM.png]

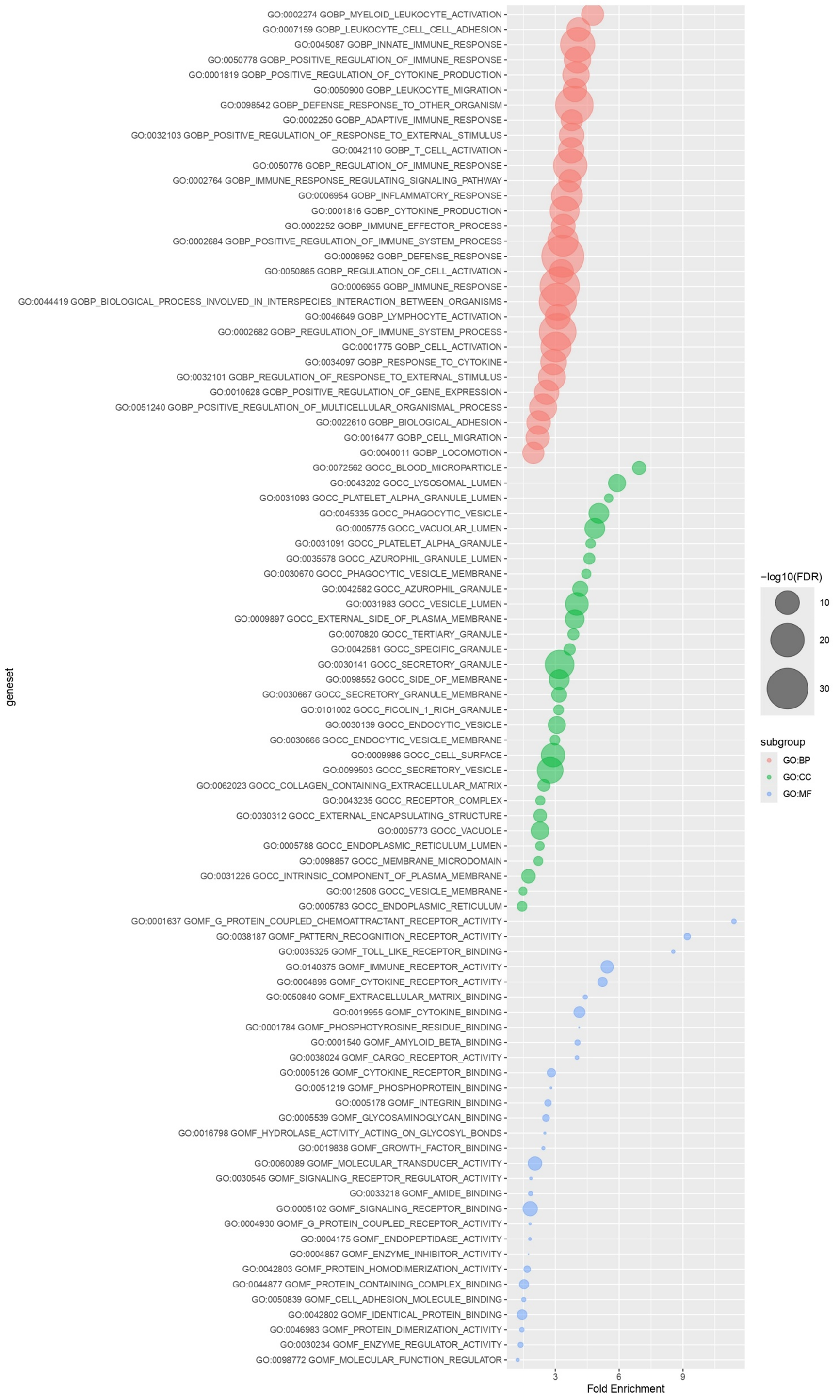

Supplement: Supplementary file 5 — Supplementary SFig. 5: Downregulated DEGs after cap59 infection of mouse brain were enriched for terms related to the immune cells and response. Bubble plots of GO analysis using hypergeometric tests (Bonferroni-corrected hypergeometric P < 0.05) of DEGs (FDR < 0.10) comparing saline-injected and cap59-infected mice at 7-dpi. The background gene set was all protein coding genes detected in the experiment. Any gene set with less than 5 genes, after filtering out genes not in the background, was excluded [file 12974_2025_3384_MOESM5_ESM.png]

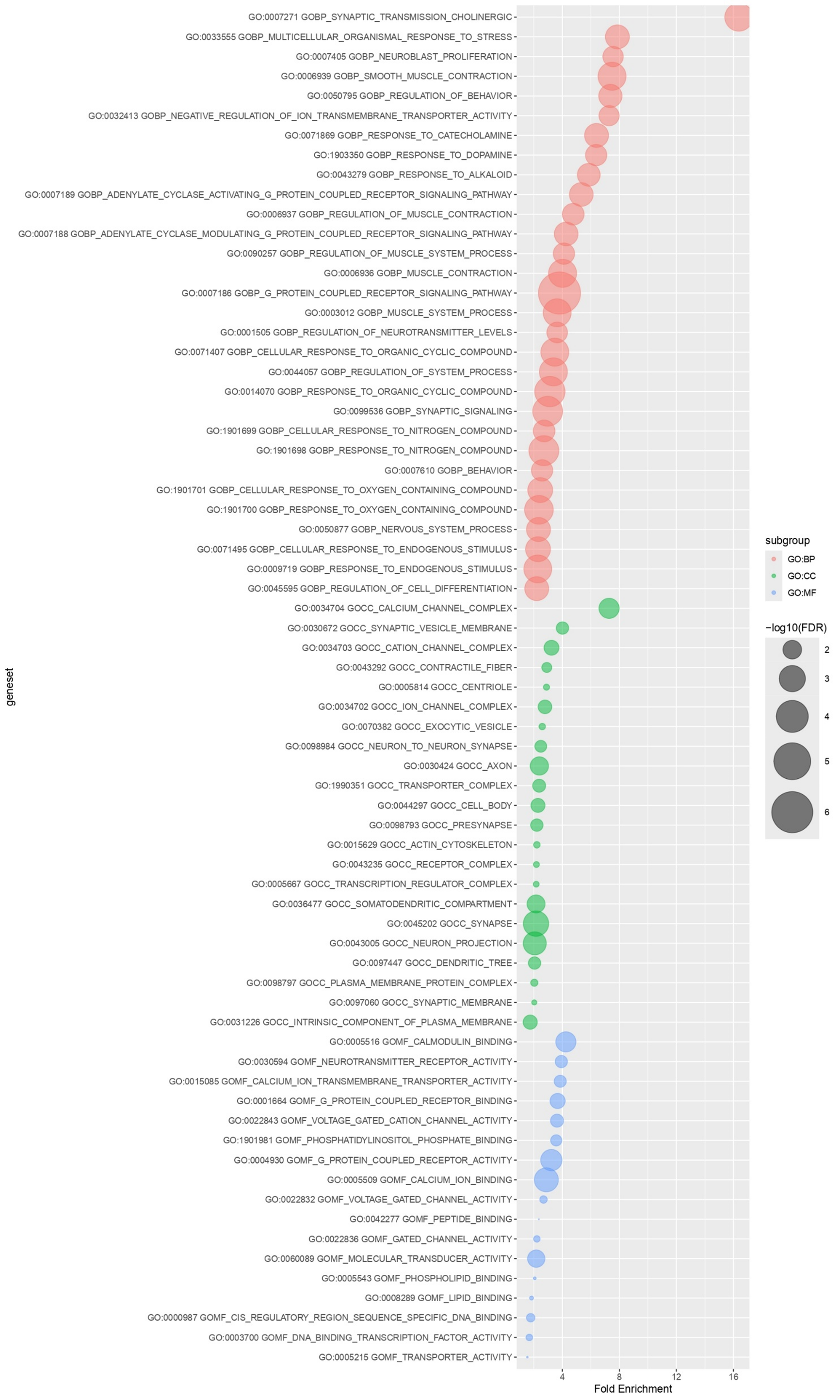

Supplement: Supplementary file 6 — Supplementary SFig. 6: Upregulated DEGs after cap59 infection of mouse brain were enriched for terms related to neuronal parts and function. Bubble plots of GO analysis using hypergeometric tests (Bonferroni-corrected hypergeometric P < 0.05) of DEGs (FDR < 0.10) comparing saline-injected and cap59-infected mice at 7-dpi. The background gene set was all protein coding genes detected in the experiment. Any gene set with less than 5 genes, after filtering out genes not in the background, was excluded [file 12974_2025_3384_MOESM6_ESM.png]

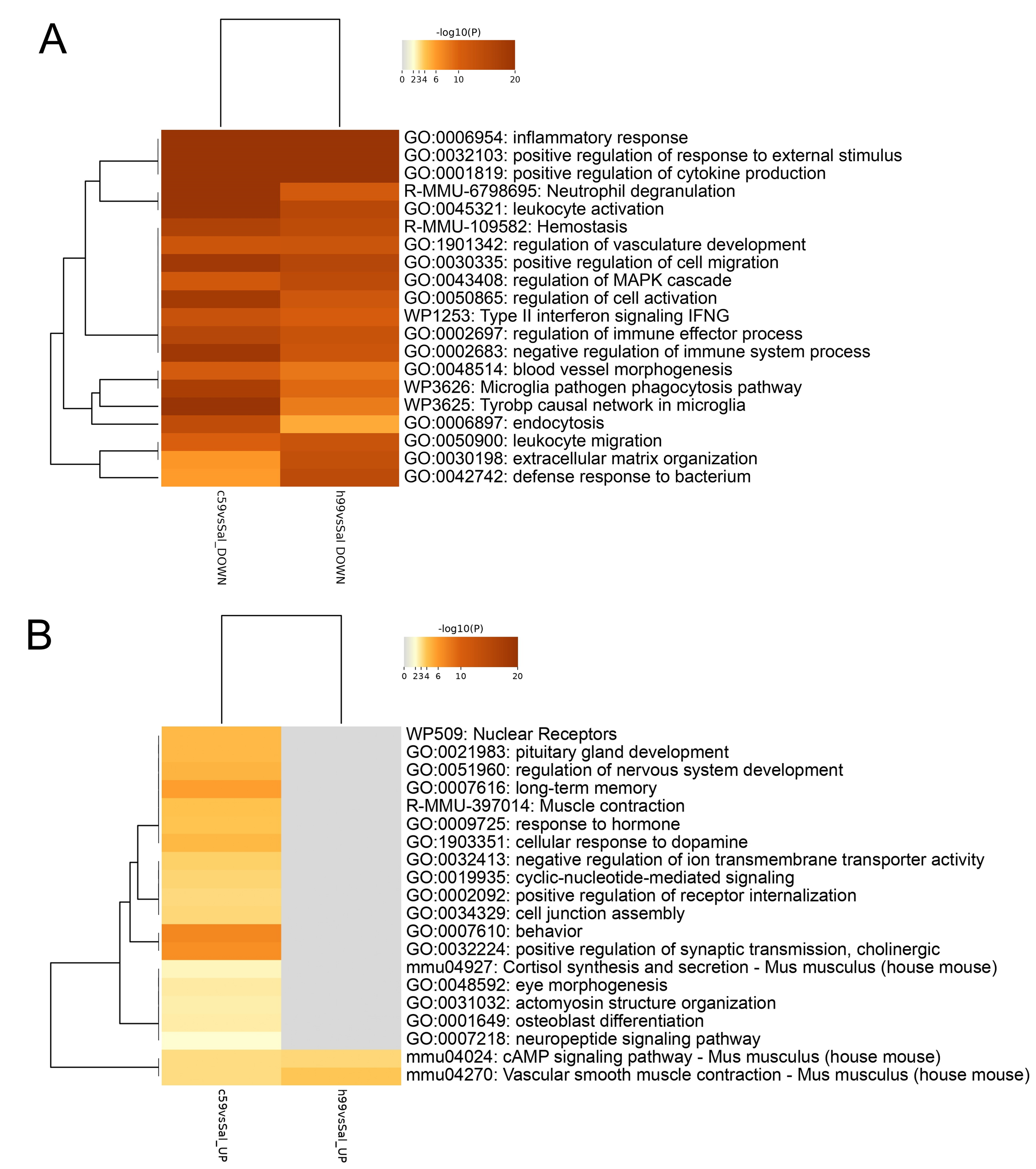

Supplement: Supplementary file 7 — Supplementary SFig. 7: Enriched ontology clusters across mouse brains injected with saline or infected with either H99 or cap59. Hierarchical clustering of top 20 overlapping enrichment ontology terms of downregulated (A) or upregulated (B) differentially expressed genes between cap59-infected vs. saline-injected and H99-infected vs. saline-injected brains. Statistically enriched terms were first identified, and accumulative hypergeometric P-values and enrichment factors were calculated and used for filtering. Significant terms were hierarchically clustered into a tree based on Kappa-statistical similarities among their gene memberships. The best P-value within each cluster was selected as its representative term. The heatmap cells are colored by their P-values; grey cells in B indicate the lack of enrichment for that term in the corresponding gene list. MCODE algorithm was then applied to this network to identify neighborhoods where proteins are densely connected. Each MCODE network is assigned a unique color. The analysis and visualization were performed using Metascape [file 12974_2025_3384_MOESM7_ESM.png]

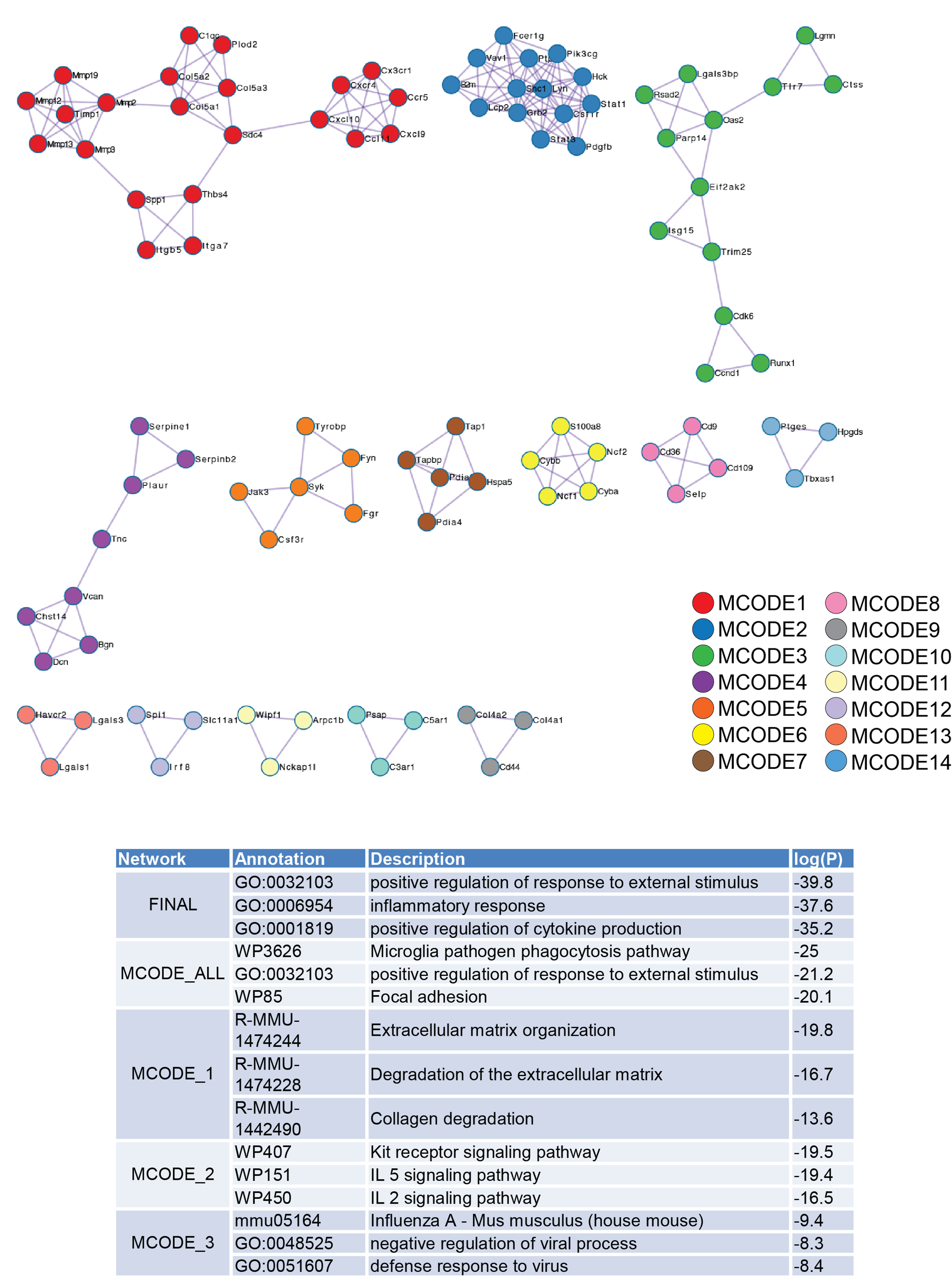

Supplement: Supplementary file 8 — Supplementary SFig. 8: Protein-protein interaction network analysis of overlapping downregulated DEGs between cap59-infected vs. saline-injected and H99-infected vs. saline-injected brains. All protein-protein interactions among downregulated DEGs were extracted and mapped into a PPI network. The network was then subjected to GO enrichment analysis to contextualize our findings. We then applied the MCODE algorithm to this network to identify neighborhoods where proteins are densely connected. Each MCODE network is assigned a different color. The table shows the top MCODE terms and top three annotations. Analysis and visualization were performed using Metascape [file 12974_2025_3384_MOESM8_ESM.png]

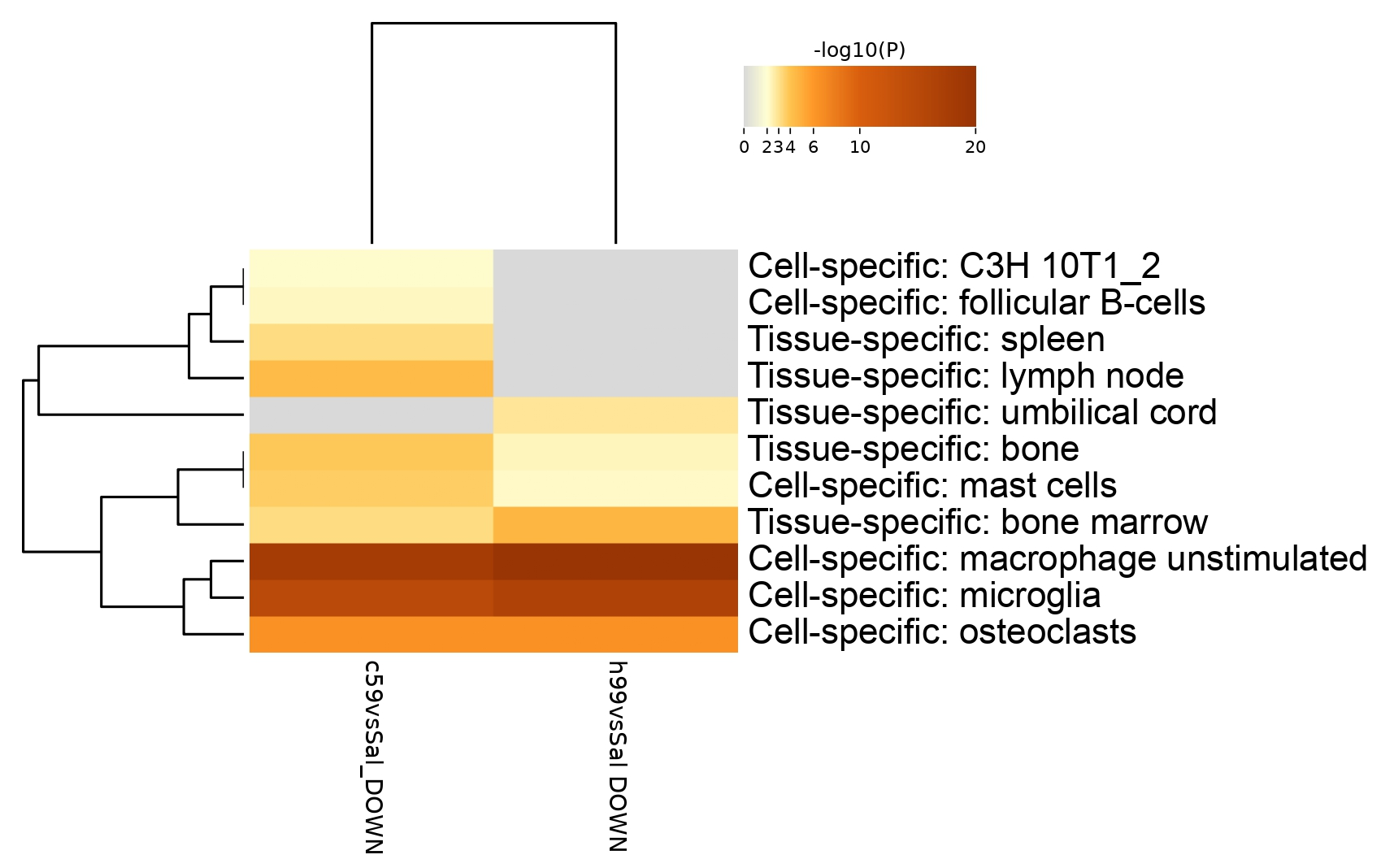

Supplement: Supplementary file 9 — Supplementary SFig. 9: Tissue/cell-specific gene signatures of downregulated DEGs between cap59-infected vs. saline-injected and H99-infected vs. saline-injected brains. The heatmap cells are colored by their P-values; grey cells indicate the lack of enrichment for that term in the corresponding gene list. Analysis and visualization were performed using Metascape (PaGenBase tool) [file 12974_2025_3384_MOESM9_ESM.png]
