# Supplementary material for: Active Cryptococcus neoformans glucuronoxylomannan production prevents elimination of cryptococcal CNS infection in vivo
Source: J Neuroinflammation. 2025 Mar 4;22:61. doi: 10.1186/s12974-025-03384-9 (PMC11877788; doi:10.1186/s12974-025-03384-9)
Supplement: Supplementary file 13 — Supplementary STable 4: Reagents used for the quantification of immune cells in vivo [file 12974_2025_3384_MOESM13_ESM.docx]

**STable 4.** Reagents used for the quantification of immune cells *in vivo*.

| Antigen | Clone | Fluorophore | Species | Cell type | Company | Catalog # | Dilution |
| --- | --- | --- | --- | --- | --- | --- | --- |
| CD45.2 | 104 | BV786 | Mouse anti-mouse | Leukocytes | BD Biosciences | 563686 | 1:100 |
| CD11b | M1/70 | APC-Cy7 | Rat anti-mouse | Neutrophils/Microglia | BD Biosciences | 557657 | 1:400 |
| Ly6G | 1A8 | PerCP-Cy5.5 | Rat anti-mouse | Neutrophils | BD Biosciences | 566435 | 1:100 |
| Zombie Aqua | N/A | BV510 | N/A | N/A | Biolegend | 423102 | 1:400 |
| Ly6C | HK1.4 | PE-Texas Red | Rat anti-mouse | Monocytes | Biolegend | 128044 | 1:200 |
| F4/80 | BM8 | BV711 | Rat anti-mouse | Macrophages | Biolegend | 123147 | 1:400 |
| MHC II | M5/114.15.2 | BV650 | Rat anti-mouse | Dendritic cells | Thermo Fisher | 64-5321-82 | 1:200 |
| B220 | RA3-6B2 | APC-A | Rat anti-mouse/human | B cells | Biolegend | 103212 | 1:100 |
| CD3e | 145-2C11 | PE | Armenian hamster anti-mouse | T cells | eBioscience | 12-0031-82 | 1:100 |
| CD4 | RM4-5 | BV605 | Rat anti-mouse | CD4 T cells | Biolegend | 100548 | 1:400 |
| CD8a | 53-6.7 | AF700 | Rat anti-mouse | CD8 T cells | eBioscience | 56-0081-82 | 1:200 |
